# PACS‐2 Mitigates NPSC Apoptosis and Intervertebral Disc Degeneration by Preserving MAM Integrity via the SP1/LRRK2/Mfn2 Axis

**DOI:** 10.1002/advs.202511781

**Published:** 2025-11-05

**Authors:** Liang Kang, Jiaqi Wang, Chenhao Zhao, Qiuwei Li, Zhigang Zhang, Huaqing Zhang, Chongyu Jia, Luping Zhou, Yanxin Wang, Yu Chen, Kaixuan Li, Xu Yan, Jie Fang, Haibao Wang, Dandan Wang, Pingping Su, Jingyu Zhang, Zhiwei Chen, Renjie Zhang, Cailiang Shen

**Affiliations:** ^1^ Department of Orthopedics and Spine Surgery The First Affiliated Hospital of Anhui Medical University Hefei 230022 China; ^2^ Laboratory of Spinal and Spinal Cord Injury Regeneration and Repair The First Affiliated Hospital of Anhui Medical University Hefei 230022 China; ^3^ Anhui Province Research Center for The Clinical Application of Digital Medical Technology The First Affiliated Hospital of Anhui Medical University Hefei 230022 China; ^4^ Department of Radiology The First Affiliated Hospital of Anhui Medical University Hefei 230022 China; ^5^ Department of Neurology The First Affiliated Hospital of Anhui Medical University Hefei 230022 China; ^6^ Laboratory Animal Core of Institute of Health and Medicine Hefei Comprehensive National Science Center Hefei 230031 China

**Keywords:** apoptosis, intervertebral disc degeneration, mitochondria‐associated endoplasmic reticulum membrane, nucleus pulposus–derived stem cells, phosphofurin acidic cluster sorting protein 2

## Abstract

Intervertebral disc (IVD) degeneration (IDD) is a leading cause of lower back pain, and the application of nucleus pulposus–derived stem cells (NPSCs) holds promise for regenerative treatment. However, the harsh microenvironment of degenerative IVDs increases apoptosis in endogenous and transplanted NPSCs, limiting the effectiveness of NPSC‐based therapies. Mitochondria‐associated ER membrane (MAM) facilitates communication between mitochondria and ER and is critical for cellular homeostasis. PACS‐2 is a central regulator of MAM homeostasis. It is found that MAM structure is disrupted in degenerative human and rat IVDs and in NPSCs exposed to an acidic environment, coinciding with reduced PACS‐2 expression and increased apoptosis. In addition, Pacs‐2 knockout mice with IDD displayed accelerated degeneration, accompanied by the exacerbation of ER stress, mitochondrial dysfunction, and apoptosis. Mechanistically, PACS‐2 suppresses phosphorylation and nuclear translocation of the transcription factor SP1, thereby downregulating its downstream target LRRK2. This reduces LRRK2‐mediated ubiquitination and degradation of Mfn2 through the JNK pathway, preserving MAM integrity and promoting NPSC survival. In vivo, transplantation of Pacs‐2–overexpressing NPSCs improved cell survival and enhanced IVD repair in a degenerative model. These findings demonstrate that PACS‐2 supports NPSC‐mediated IVD regeneration by maintaining MAM integrity via the SP1/LRRK2/Mfn2 axis, offering potential therapeutic targets for IDD.

## Graphical abstract

This study investigates the role of PACS‐2 in IDD, demonstrating that it suppresses the nuclear translocation of SP1, thereby downregulating its downstream target LRRK2. This reduces LRRK2‐mediated ubiquitination and degradation of Mfn2 through the JNK pathway, preserving MAM integrity and promoting NPSC survival. Transplantation of PACS‐2‐overexpressing NPSCs enhances cell survival and promotes IVD repair, offering potential therapeutic targets for IDD.

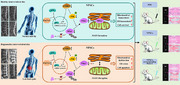

## 1 Introduction

Low back pain (LBP) is a significant cause of disability and functional limitation among adults worldwide, presenting a considerable economic and healthcare burden.^[^ ^1^ ^]^ By 2050, it is estimated that over 800 million individuals globally will experience LBP.^[^ ^2^ ^]^ Intervertebral disc (IVD) degeneration (IDD) is recognized as the primary cause of LBP. Despite advances in the clinical management of IDD, the overall clinical results remain unsatisfactory because current treatments mainly relieve symptoms rather than address the underlying causes to halt or reverse disease progression.^[^ ^3^ ^]^ Additionally, surgical complications adversely affect patients’ quality of life. Stem cell‐based therapies hold promise for the treatment of various diseases, including IDD.^[^ ^4^ ^]^ Previous studies, including those by our group, have shown that IVDs contain nucleus pulposus (NP)‐derived stem cells (NPSCs), which represent a promising cell source for cell therapy and tissue engineering aimed at IVD repair and regeneration.^[^ ^5^, ^6^ ^]^ However, the hostile microenvironment in degenerated IVDs causes a progressive decline in the quality and quantity of NPSCs, ultimately leading to the failure of stem cell‐based IVD repair.^[^ ^7^ ^]^

The IVD is the largest avascular tissue in the human body and depends mainly on glycolysis for energy metabolism. During IDD, decreased permeability of the cartilaginous endplate leads to the accumulation of metabolic byproducts, such as lactic acid, resulting in an acidic microenvironment. This acidosis disrupts the balance of matrix metabolism and promotes apoptosis of both endogenous disc cells and transplanted cells from cell therapies.^[^ ^8^ ^]^ Our recent studies further demonstrate that acidosis accelerates NPSC apoptosis.^[^ ^9^ ^]^ Nevertheless, approaches to protect NPSCs from acid‐induced cell death require further investigation.

Emerging evidence highlights the importance of endoplasmic reticulum (ER) stress and mitochondrial dysfunction in the pathogenesis of IDD.^[^ ^10^, ^11^ ^]^ The identification of the mitochondria‐associated ER membrane (MAM) highlights the close functional and physical association between the mitochondria and ER.^[^ ^12^ ^]^ MAMs serve as contact sites that play a crucial role in maintaining cellular homeostasis by regulating mitochondrial quality control, ER stress, calcium homeostasis, lipid metabolism, autophagy, and apoptosis. However, the role of MAMs in IDD is not well defined. Specific proteins localized to the MAM are essential for maintaining its structure and function, and research on these protein advances understanding of MAM physiology and disease mechanisms. Phosphofurin acidic cluster sorting protein 2 (PACS‐2) is a key MAM regulator, implicated in diseases such as obesity, Alzheimer's disease, and diabetic nephropathy.^[^ ^13^ ^]^ Xue et al.^[^ ^14^ ^]^ reported that PACS‐2 overexpression stabilizes MAMs and reduces apoptosis in renal tubular epithelial cells. In contrast, Yu et al.^[^ ^15^ ^]^ found that PACS‐2 knockout impairs MAM formation and reduces apoptosis in endothelial cells exposed to oxidized low‐density lipoprotein. Because PACS‐2‐mediated MAM homeostasis may play different roles across cell types and disease contexts, and its function in IDD is unknown, it is important to determine whether modulating PACS‐2 levels in NPSCs can help these cells adapt to the adverse microenvironment of degenerated IVDs.

In this study, we found that NP tissues from IDD patients and puncture‐induced rat IDD models, as well as NPSCs in an acid‐induced in vitro degeneration model, exhibited reduced PACS‐2 expression, disrupted MAM integrity, and increased apoptosis. Pacs‐2 gene knockout (Pacs‐2^–/–^) mice developed more severe disc degeneration, with the exacerbation of ER stress, mitochondrial dysfunction, and apoptosis. In vitro, PACS‐2 overexpression in NPSCs reversed the deleterious effects of acid exposure. Mechanistically, PACS‐2 inhibited the phosphorylation and nuclear translocation of specificity protein 1 (SP1), reducing the expression of its downstream target, leucine‐rich repeat kinase 2 (LRRK2). This, in turn, suppressed c‐Jun N‐terminal kinase (JNK) pathway‐mediated ubiquitination and degradation of mitofusin‐2 (Mfn2), ultimately preserving MAM integrity and promoting NPSC survival. Furthermore, transplantation of PACS‐2‐overexpressing NPSCs into degenerated IVDs in a rat model improved cell survival and repair function. These findings indicate that PACS‐2 promotes stem cell‐based IVD repair and regeneration, providing a new therapeutic strategy for IDD.

## 2 Results

### 2.1 PACS‐2 Expression was Downregulated and MAM Integrity was Reduced in Degenerative NP Tissues and Acid‐Treated NPSCs

**Figure 1:**
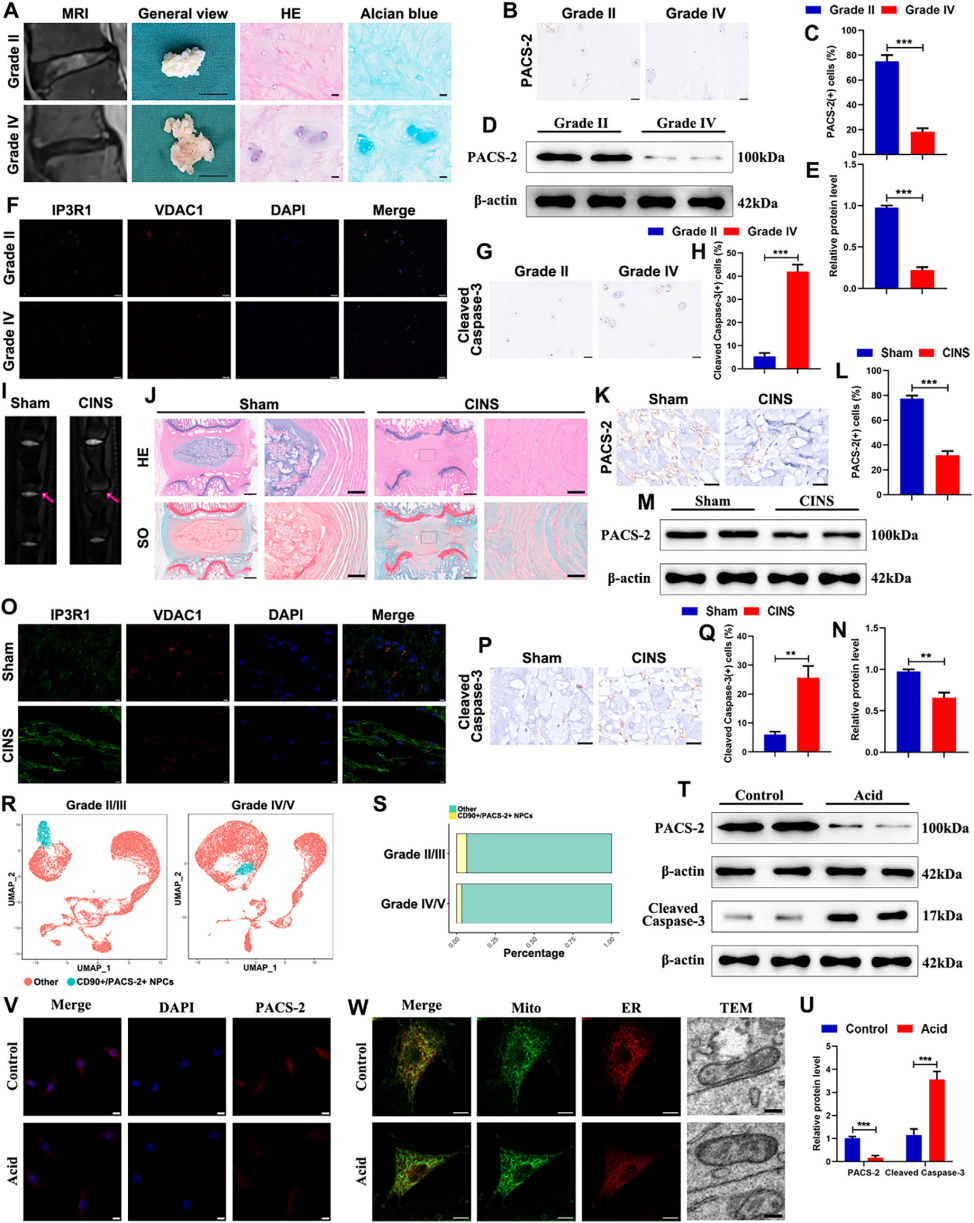
Impaired PACS‐2 expression and MAM integrity in degenerative NP tissues of humans and rats, as well as acid‐treated NPSCs. A) Representative MRI images, General views (Scale bar: 1 cm), HE staining images(Scale bar: 25 µm), and Alcian blue staining images (Scale bar: 25 µm) of human IVD tissues with Grade II and Grade IV degeneration. B,C) Representative immunohistochemical staining images of PACS‐2 in human NP tissues and the quantitative analysis (n = 5). Scale bar: 25 µm. D,E) Representative western blot images and quantitative analysis of PACS‐2 in human NP tissues (n = 5). F) Representative immunofluorescence double staining images of IP3R1 and VDAC1 in human NP tissues. Scale bar: 20 µm. G,H) Representative immunohistochemical staining images of Cleaved caspase‐3 in human NP tissues and the quantitative analysis (n = 5). Scale bar: 25 µm. I) Representative MRI images of rat tails. J) Representative HE staining images and SO staining images of rat IVD tissues. Scale bar: left panel, 500 µm; right panel, 100 µm. K,L) Representative immunohistochemical staining images of PACS‐2 in rat NP tissues and the quantitative analysis (n=5). Scale bar: 25 µm. M,N) Representative western blot images and quantitative analysis of PACS‐2 in rat NP tissues (n = 5). O) Representative immunofluorescence double staining images of IP3R1 and VDAC1 in rat NP tissues. Scale bar: 5 µm. P,Q) Representative immunohistochemical staining images of Cleaved caspase‐3 in rat NP tissues and the quantitative analysis (n=5). Scale bar: 25 µm. R) UMAP visualization of cell populations distribution between mild IDD (Pfirrmann II/III) group and severe IDD (Pfirrmann IV/V) group. S) Percentage of different cell types in mild and severe IDD group. T,U) Representative western blot images and quantitative analysis of PACS‐2 and Cleaved caspase‐3 in human NPSCs exposed to acid medium of pH 6.5 for 24 h (n = 3). V) Representative immunofluorescence staining images of PACS‐2 in the acid‐treated NPSCs (n=3). Scale bar: 10 µm. W) Representative fluorescence confocal images with Mito‐Tracker (green) and ER‐Tracker (red) staining in the acid‐treated NPSCs. Scale bar: 10 µm. Representative TEM images of NPSCs. Scale bar: 200 nm. Data are presented as mean ± SD. ^*^ *p* < 0.05, ^**^ *p* < 0.01, and ^***^ *p* < 0.001.

To investigate changes in PACS‐2‐associated MAM homeostasis during the development of IDD, NP tissues were collected from patients with varying degrees of IDD according to the Pfirrmann grading system (**Figure** **1**A). Compared to Pfirrmann II NP tissues, Pfirrmann IV NP tissues showed volume reduction, decreased elasticity, increased cell clustering, and reduced extracellular matrix content (Figure 1A). Western blot analysis and immunohistochemical staining demonstrated lower PACS‐2 levels in Pfirrmann IV NP tissues relative to Pfirrmann II NP tissues (Figure 1B–E). Immunofluorescence double staining with antibodies against inositol 1,4,5‐trisphosphate receptor type 1 (IP3R1) and voltage‐dependent anion channel 1 (VDAC1) was conducted to assess MAM formation. Reduced MAM formation was observed in Pfirrmann IV NP tissues compared with Pfirrmann II NP tissues (Figure 1F). In addition, the apoptosis level was evaluated by measuring cleaved caspase‐3 expression through immunohistochemical staining. The level of cleaved caspase‐3 was increased in Pfirrmann IV NP tissues compared to Pfirrmann II NP tissues (Figure 1G,H), which contrasted with the decrease in PACS‐2 expression and disruption of MAM integrity.

To verify these findings, a rat coccygeal IVD needle stab (CINS) model was established. Preliminary characterization using magnetic resonance imaging (MRI) indicated that disc degeneration in the CINS group was more severe than in the sham group (Figure 1I), with a higher Pfirrmann grade (Figure S1A, Supporting Information). Hematoxylin and Eosin (HE) and Safranin O‐Fast Green (SO) staining revealed a decrease in NP volume and disruption of the boundary between the annulus fibrosus and NP in the CINS group (Figure 1J). Quantitative histological scoring indicated higher scores in the CINS group compared to the sham group (Figure S1B, Supporting Information), confirming successful model establishment. Consistent with the findings in human tissues, immunohistochemical staining, immunofluorescence double staining, and western blot analysis showed reduced PACS‐2 expression, decreased MAM formation, and increased apoptosis in the CINS group (Figure 1K–Q).

Single‐cell RNA sequencing (scRNA‐seq) offers important insight into IDD mechanisms by enabling the analysis of individual cell populations. Publicly available scRNA‐seq data (GSE165722), which include NP tissues from patients with varying degrees of degeneration, were analyzed. CD90‐positive NP cells (NPCs) were previously identified as progenitor cells based on scRNA‐seq analysis, flow cytometry, and multipotency assays.^[^ ^16^ ^]^ Further analysis revealed that the proportion of CD90+PACS‐2+ NPCs was higher in mild IDD (Pfirrmann II/III) than in severe IDD (Pfirrmann IV/V) (Figure 1R,S), suggesting that loss of CD90+PACS‐2+ NPCs may be associated with IDD progression. To further investigate the role of PACS‐2‐related MAM homeostasis, stem cells isolated from human NP tissue were characterized by spindle shape, spiral growth pattern, expression of stem cell surface markers, and adipogenic, chondrogenic, and osteogenic differentiation potential (Figure S1C–E, Supporting Information), confirming their identity as NPSCs. As NPSCs in degenerated IVDs experience an acidic microenvironment, NPSCs were exposed to acidic conditions to simulate IDD. Western blot analysis showed that acid‐treated NPSCs had reduced PACS‐2 expression and increased cleaved caspase‐3 levels (Figure 1T,U). Immunofluorescence staining confirmed decreased PACS‐2 in acid‐treated NPSCs (Figure 1V). Transmission electron microscopy (TEM) and immunofluorescence analysis indicated reduced MAM formation under acidic conditions (Figure 1W). Together, these data indicate that reduced PACS‐2 expression and disrupted MAM structure are characteristic of IDD and are associated with NPSC apoptosis.

### 2.2 Pacs‐2 Deletion Accelerated IDD Progression in Mice

**Figure 2:**
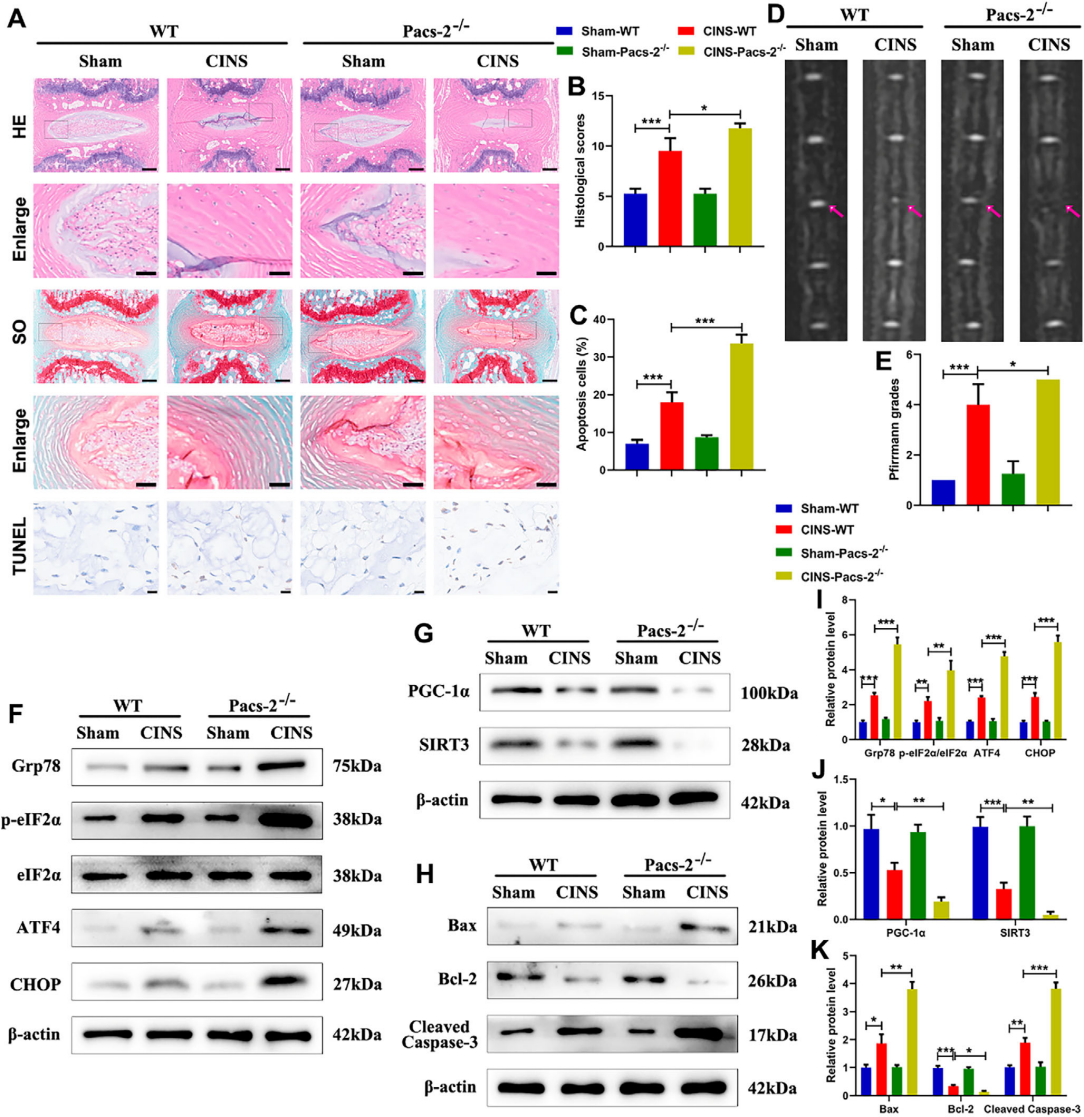
IDD‐related degenerative changes, ER stress, mitochondrial dysfunction, and apoptosis are exacerbated in Pacs‐2 gene knockout mice. A) Representative HE staining images (Scale bar: upper panel, 200 µm; lower panel, 50 µm), SO staining images (Scale bar: upper panel, 200 µm; lower panel, 50 µm), and TUNEL staining images (Scale bar: 10 µm) of mouse IVD tissues. B) Histological scores of mouse IVD tissues (n = 5). C) The proportion of apoptotic cells calculated as the percentage of TUNEL‐positive cells (n = 5). D) Representative MRI images of mouse tails. E) The MRI Pfirrmann grade analysis of the mouse tail IVDs (n = 5). F) Representative western blot images of Grp78, p‐eIF2α, eIF2α, ATF4, and CHOP in mouse NP tissues. G) Representative western blot images of PGC‐1α and SIRT3 in mouse NP tissues. H) Representative western blot images of Bax, Bcl‐2, and Cleaved caspase‐3 in mouse NP tissues. I) Quantitative analysis of the protein expression levels in (F). J) Quantitative analysis of the protein expression levels in (G). K) Quantitative analysis of the protein expression levels in (H) (n = 3). Data are presented as mean ± SD. ^*^ *p* < 0.05, ^**^ *p* < 0.01, and ^***^ *p* < 0.001.

To further elucidate the role of PACS‐2 in IDD progression, a CINS model was generated using Pacs‐2^−/−^ and wild‐type (WT) mice. Western blot analysis verified efficient Pacs‐2 knockout, with undetectable expression in NP tissues from Sham and CINS Pacs‐2^−/−^ mice (Figure S2, Supporting Information). MRI, HE, SO, and TUNEL staining showed that CINS WT mice exhibited severe degenerative changes and increased apoptosis compared to Sham WT mice, and these changes were further exacerbated in CINS Pacs‐2^−/−^ mice (**Figure** **2**A–E). Western blot analysis was performed to assess markers of ER stress, mitochondrial function, and apoptosis. CINS WT mice showed elevated levels of ER stress markers (Glucose‐regulated protein 78 (Grp78), phosphorylated eukaryotic initiation factor 2α (p‐eIF2α)/eIF2α, activating transcription factor 4 (ATF4), and C/EBP homologous protein (CHOP), which were further increased in CINS Pacs‐2^−/−^ mice (Figure 2F–I). Mitochondrial function, assessed by peroxisome proliferator‐activated receptor gamma coactivator 1‐alpha (PGC‐1α) and sirtuin 3 (SIRT3) expression, was impaired in CINS WT mice and further aggravated in CINS Pacs‐2^−/−^ mice (Figure 2G–J). Increased apoptosis was evidenced by decreased Bcl‐2 and increased Bax and cleaved caspase‐3 in CINS WT mice, with further enhancement in CINS Pacs‐2^−/−^ mice (Figure 2H–K). Collectively, these findings support a protective role of PACS‐2 against IDD progression.

### 2.3 Overexpression of PACS‐2 Prevented ER Stress, Mitochondrial Dysfunction, and Apoptosis by Enhancing MAM Formation in Acid‐Treated Human NPSCs

**Figure 3:**
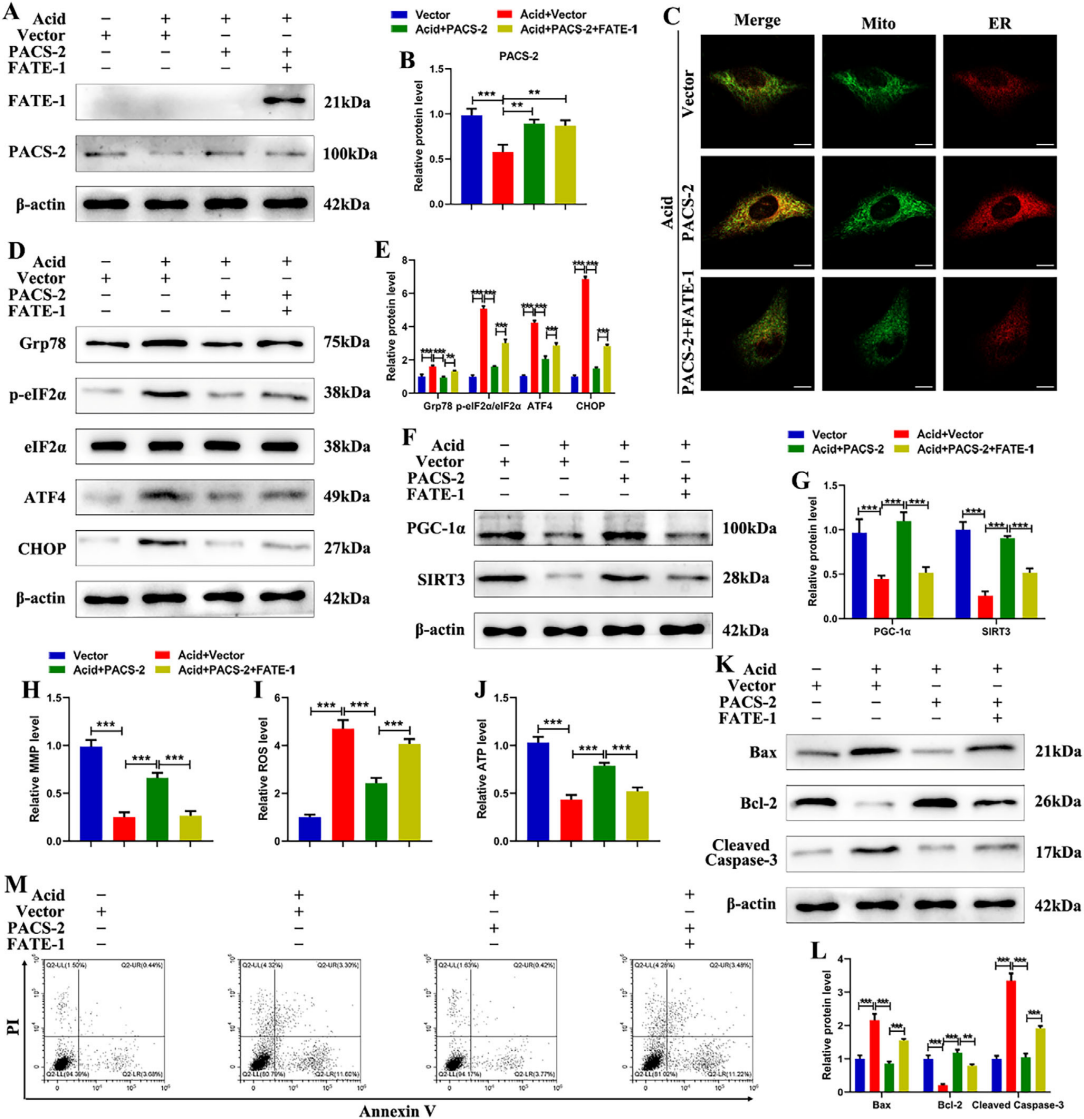
Effects of PACS‐2 overexpression and MAM integrity on acid‐induced NPSC injury. A) Representative western blot images of FATE‐1 and PACS‐2 in NPSCs treated with PACS‐2 plasmid or co‐treated with PACS‐2 plasmid and FATE‐1 plasmid under acidic conditions. B) Quantitative analysis of the protein expression levels in (A) (n = 3). C) Representative fluorescence confocal images with Mito‐Tracker (green) and ER‐Tracker (red) staining in NPSCs. Scale bar: 10 µm. D,E) Representative western blot images and quantitative analysis of Grp78, p‐eIF2α, eIF2α, ATF4, and CHOP in NPSCs (n = 3). F,G) Representative western blot images and quantitative analysis of PGC‐1α and SIRT3 in NPSCs (n=3). H) Mitochondrial membrane potential was determined by using JC‐1 (n = 3). I) ROS generation was assessed using DCFH‐DA (n = 3). J) ATP content was detected using an ATP assay kit (n = 3). K,L) Representative western blot images and quantitative analysis of Bax, Bcl‐2, and Cleaved caspase‐3 in NPSCs (n = 3). M) Flow cytometry analysis of NPSC apoptosis rate. Data are presented as mean ± SD. ^*^ *p* < 0.05, ^**^ *p* < 0.01, and ^***^ *p* < 0.001.

To clarify the role of MAM homeostasis in PACS‐2‐mediated resistance to IDD, the expression levels of PACS‐2 and fetal and adult testis expressed 1 (FATE‐1) were modulated either individually or simultaneously in NPSCs. Western blot analysis confirmed that transfection with PACS‐2 plasmid alone or co‐transfection with FATE‐1 plasmid successfully increased the respective protein levels in NPSCs treated with (**Figure** **3**A,B) or without (Figure S3A,B, Supporting Information) acidic condition. FATE‐1 was employed as an uncoupling agent for MAM due to its capacity to dissociate the ER–mitochondria interface. Immunofluorescence double staining with Mito‐tracker and ER‐tracker revealed that FATE‐1 co‐treatment abrogated the PACS‐2‐mediated enhancement of MAM integrity in acid‐treated NPSCs (Figure 3C). Western blot analysis demonstrated that PACS‐2 overexpression partially inhibited the acid‐induced upregulation of ER stress markers Grp78, p‐eIF2α/eIF2α, ATF4, and CHOP, whereas these effects were diminished by co‐expression of FATE‐1 (Figure 3D,E). In acid‐treated NPSCs, PACS‐2 overexpression attenuated the reduction in PGC‐1α and SIRT3 protein levels, the loss of mitochondrial membrane potential (MMP), and the decrease in ATP production. PACS‐2 overexpression also suppressed the acid‐induced increase in reactive oxygen species (ROS) levels (Figure 3F–J). Consistent with improved mitochondrial function, PACS‐2 overexpression reduced apoptosis in NPSCs under acidic conditions, as shown by western blot and flow cytometry (Figure 3K–M). The protective effects of PACS‐2 were attenuated by co‐overexpression of FATE‐1 (Figure 3F–M). These data indicate that PACS‐2 supports ER and mitochondrial homeostasis and promotes NPSC survival by restoring MAM integrity in the setting of acid‐induced injury.

### 2.4 PACS‐2 Preserved MAM Integrity and Promoted NPSCs Survival Through LRRK2/Mfn2 Pathway

To clarify the mechanism by which PACS‐2 regulates MAM integrity, RNA sequencing was performed on NPSCs transfected with small interfering RNA (siRNA) targeting PACS‐2 (si‐PACS‐2) under acidic conditions. As shown in the heatmap and volcano plot, 1452 differentially expressed genes were identified between the acid+scrambled siRNA (si‐Scr) group and the acid+si‐PACS‐2 group, with 692 genes upregulated and 760 genes downregulated in the si‐PACS‐2 group (Figure S4A,B, Supporting Information). Gene ontology (GO) analysis indicated that these differentially expressed genes were enriched in biological processes such as positive regulation of mitochondrial depolarization, mesenchymal cell apoptosis, positive regulation of ROS biosynthetic process, and intracellular calcium ion homeostasis (Figure S4C, Supporting Information). These findings are consistent with the biological role of PACS‐2 in NPSCs.

**Figure 4:**
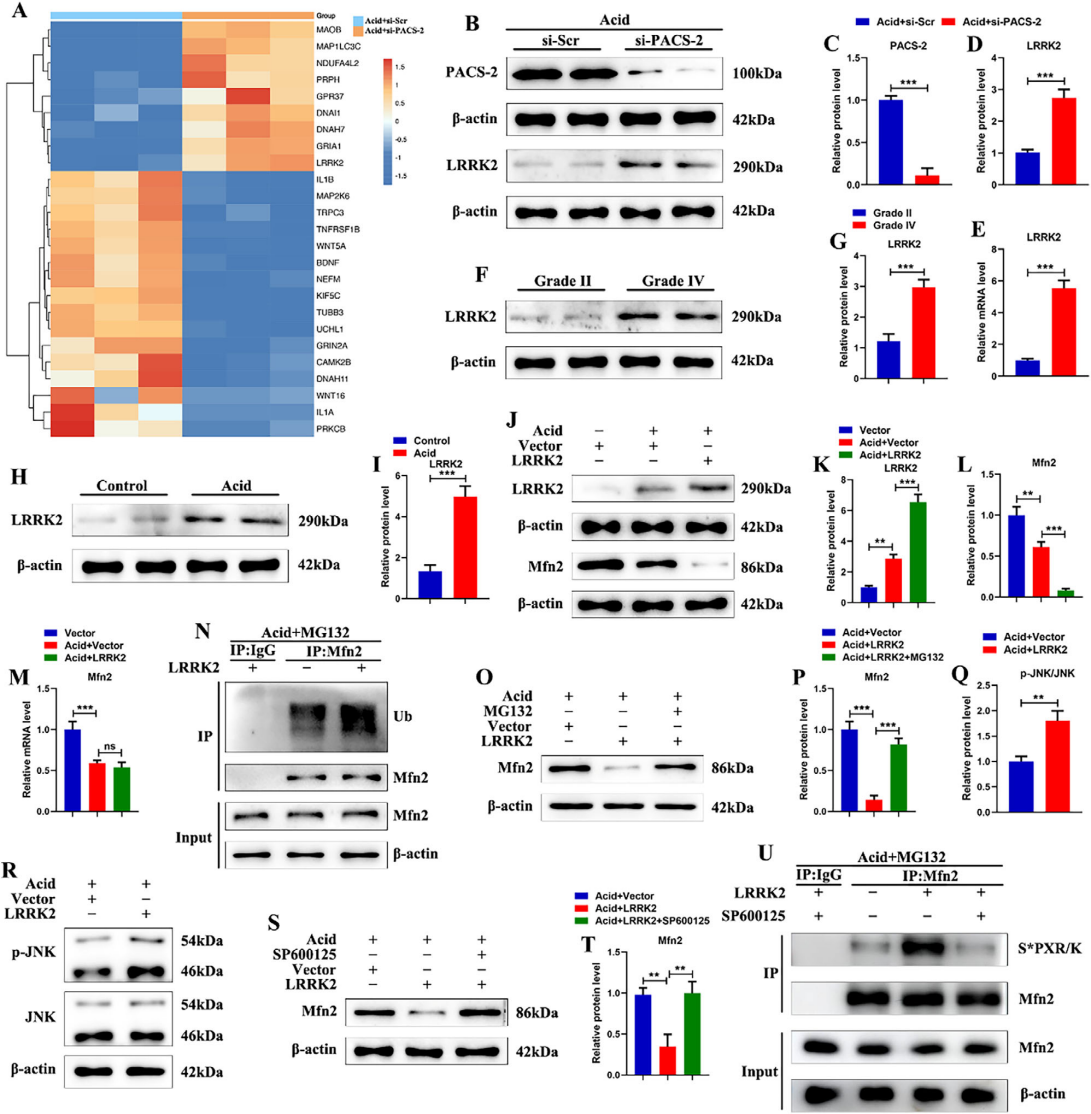
The increase in LRRK2 expression induced by PACS‐2 knockdown is related to the decrease in Mfn2 expression. A) RNA sequencing shows that LRRK2 is a significantly upregulated gene in NPSCs transfected with si‐PACS‐2 under acidic conditions. B–D) Representative western blot images and quantitative analysis of PACS‐2 and LRRK2 in NPSCs treated with si‐Scr and si‐PACS‐2 under acidic conditions (n = 3). E) The relative mRNA levels of LRRK2 in NPSCs treated with si‐Scr and si‐PACS‐2 under acidic conditions (n = 3). F,G) Representative western blot images and quantitative analysis of LRRK2 in human NP tissues with Grade II and Grade IV degeneration. H,I) Representative western blot images and quantitative analysis of LRRK2 in human NPSCs under acidic conditions (n = 3). J,K,L) Representative western blot images and quantitative analysis of LRRK2 and Mfn2 in NPSCs treated with LRRK2 plasmid and control vector under acidic conditions (n = 3). M) The relative mRNA levels of Mfn2 in NPSCs treated with LRRK2 plasmid and control vector under acidic conditions (n = 3). N) Measurement of ubiquitination of Mfn2 after immunoprecipitation with an anti‐Mfn2 antibody through western blot analysis with antibodies recognizing ubiquitin or Mfn2 (n = 3). O,P) Representative western blot images and quantitative analysis of Mfn2 in LRRK2 plasmid‐transfected NPSCs under acidic conditions, with or without MG132 treatment (20 µM) for 2 h (n = 3). Q,R) Representative western blot images and quantitative analysis of phosphorylated JNK (p‐JNK) and total JNK in NPSCs treated with LRRK2 plasmid and control vector under acidic conditions (n = 3). S,T) Representative western blot images and quantitative analysis of Mfn2 in LRRK2 plasmid‐transfected NPSCs under acidic conditions, with or without JNK inhibitor SP600125 treatment (10 µM) for 2 h (n = 3). U) Immunoprecipitated endogenous Mfn2 was immunoblotted with antibody recognizing either S*PXR/K (S* = Phospho‐Serine) or Mfn2 (n = 3). Data are presented as mean ± SD. ns, not significant, ^*^ *p* < 0.05, ^**^ *p* < 0.01, and ^***^ *p* < 0.001.

The expression of LRRK2, a multifunctional protein known to promote Mfn2 degradation, was significantly increased in the si‐PACS‐2 group compared with the si‐Scr group under acidic conditions (**Figure** **4**A). qRT‐PCR and western blot analysis confirmed that PACS‐2 knockdown elevated both the protein and mRNA levels of LRRK2 in acid‐treated NPSCs (Figure 4B–E). Furthermore, western blot analysis demonstrated that LRRK2 expression was upregulated in degenerative NP tissues and acid‐treated NPSCs, showing an opposite trend to PACS‐2 expression (Figure 4F–I). Mfn2 is a critical regulator of MAM formation. Under cellular stress, Mfn2 is phosphorylated by JNK on Ser27, leading to its ubiquitination and degradation.^[^ ^17^ ^]^ Given that LRRK2 promotes Mfn2 degradation and is classified as a member of the mixed lineage kinase (MLK) subfamily of mitogen‐activated protein kinase kinase kinases (MAPKKKs), which can activate JNK,^[^ ^18^, ^19^ ^]^ we explored whether PACS‐2 regulates Mfn2 levels through the LRRK2/JNK pathway, thereby affecting MAM integrity.

The effect of LRRK2 on Mfn2 was first assessed in acid‐treated NPSCs. Overexpression of LRRK2 reduced Mfn2 protein levels, but did not alter Mfn2 mRNA levels (Figure 4J–M). Additional experiments demonstrated that LRRK2 overexpression increased the ubiquitination of Mfn2 in acid‐treated NPSCs (Figure 4N). Treatment with the proteasome inhibitor MG132 reversed the reduction in Mfn2 protein levels caused by LRRK2 overexpression (Figure 4O,P). Western blot analysis showed that LRRK2 overexpression increased JNK phosphorylation in NPSCs under acidic conditions (Figure 4Q,R). The addition of SP600125, a JNK inhibitor, significantly reversed the reduction in Mfn2 levels induced by LRRK2 (Figure 4S,T). To confirm Mfn2 Ser27 phosphorylation, we immunoprecipitated Mfn2 and performed immunoblotting using antibody that recognize S*PXR/K (S* represents Phospho‐Serine). We observed that overexpression of LRRK2 induced phosphorylation of Mfn2 Ser27, but this was significantly inhibited by the addition of SP600125 (Figure 4U). These findings suggest that LRRK2 promotes Mfn2 degradation via a JNK‐dependent pathway.

**Figure 5:**
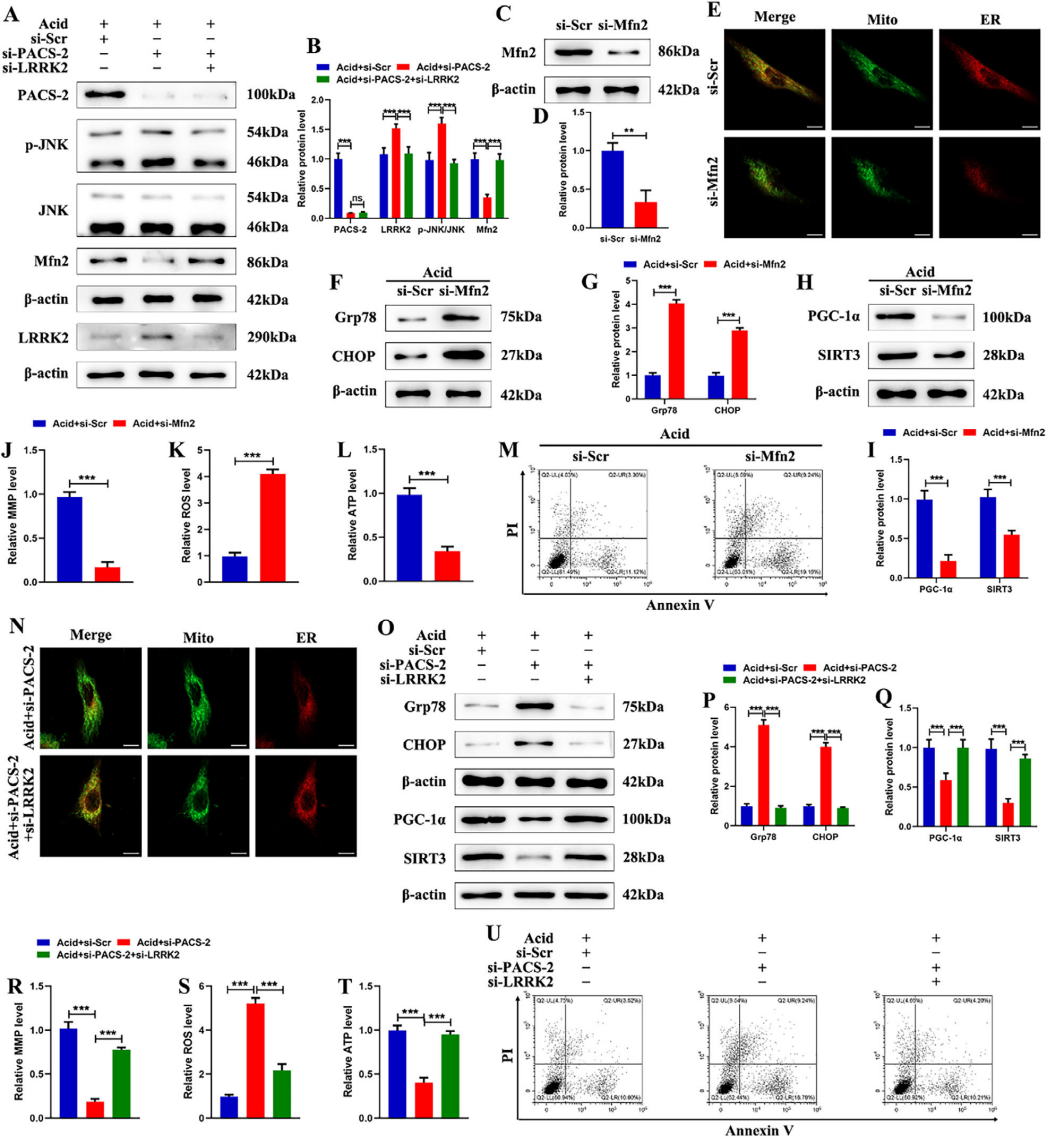
PACS‐2 promotes MAM formation and NPSC survival by upregulating Mfn2 levels through the inhibition of the LRRK2/JNK pathway. A) Representative western blot images of PACS‐2, LRRK2, p‐JNK, total JNK, and Mfn2 in NPSCs treated with si‐PACS‐2 or co‐treated with si‐PACS‐2 and si‐LRRK2 under acidic conditions. B) Quantitative analysis of the protein expression levels in (A) (n = 3). C,D) Representative western blot images and quantitative analysis of Mfn2 in NPSCs treated with si‐Scr and si‐Mfn2 (n = 3). E) Representative fluorescence confocal images with Mito‐Tracker (green) and ER‐Tracker (red) staining in NPSCs treated with si‐Scr and si‐Mfn2. F,G) Representative western blot images and quantitative analysis of Grp78 and CHOP in NPSCs treated with si‐Scr and si‐Mfn2 under acidic conditions (n = 3). H,I) Representative western blot images and quantitative analysis of PGC‐1α and SIRT3 in NPSCs (n = 3). J) Mitochondrial membrane potential was determined by using JC‐1 (n = 3). K) ROS generation was assessed using DCFH‐DA (n = 3). L) ATP content was detected using an ATP assay kit (n = 3). M) Flow cytometry analysis of NPSC apoptosis rate. N) Representative fluorescence confocal images with Mito‐Tracker (green) and ER‐Tracker (red) staining in NPSCs treated with si‐PACS‐2 or co‐treated with si‐PACS‐2 and si‐LRRK2 under acidic conditions. O–Q) Representative western blot images and quantitative analysis of Grp78 and CHOP, PGC‐1α, and SIRT3 in NPSCs treated with si‐PACS‐2 or co‐treated with si‐PACS‐2 and si‐LRRK2 under acidic conditions (n = 3). R) Mitochondrial membrane potential was determined by using JC‐1 (n = 3). S) ROS generation was assessed using DCFH‐DA (n = 3). T) ATP content was detected using an ATP assay kit (n = 3). U) Flow cytometry analysis of NPSC apoptosis rate. Data are presented as mean ± SD. ^*^ *p* < 0.05, ^**^ *p* < 0.01, and ^***^ *p* < 0.001.

To further investigate the role of the LRRK2/Mfn2 axis in PACS‐2‐mediated MAM homeostasis, si‐PACS‐2 and si‐LRRK2 were co‐transfected into human NPSCs under acidic conditions. Western blot analysis indicated that LRRK2 knockdown reversed the increased JNK phosphorylation and decreased Mfn2 levels resulting from PACS‐2 deficiency (**Figure** **5**A,B). MAM integrity relies on the interaction of ER–mitochondrial tethering proteins, with Mfn2 being a key component. Knockdown of Mfn2 impaired MAM integrity in NPSCs (Figure 5C–E), increased Grp78 and CHOP levels, reduced PGC‐1α and SIRT3 levels, decreased MMP and ATP production, and elevated ROS and apoptosis (Figure 5F–M). These results support the role of Mfn2 in maintaining MAM homeostasis and promoting NPSC survival. Immunofluorescence staining showed that LRRK2 knockdown increased MAM formation in NPSCs transfected with si‐PACS‐2 under acidic conditions (Figure 5N). Moreover, LRRK2 knockdown ameliorated PACS‐2 deficiency‐induced ER stress, mitochondrial dysfunction, and apoptosis, as shown by decreased Grp78 and CHOP, increased PGC‐1α and SIRT3, improved MMP and ATP production, and reduced ROS and apoptosis after co‐transfection with si‐LRRK2 (Figure 5O–U). Collectively, these findings indicate that PACS‐2 maintains MAM integrity and NPSC homeostasis by reducing the ubiquitination and degradation of Mfn2 through the LRRK2/JNK pathway.

### 2.5 PACS‐2 Inhibited LRRK2 Transcription by Suppressing the Nuclear Translocation of SP1 in NPSCs

**Figure 6:**
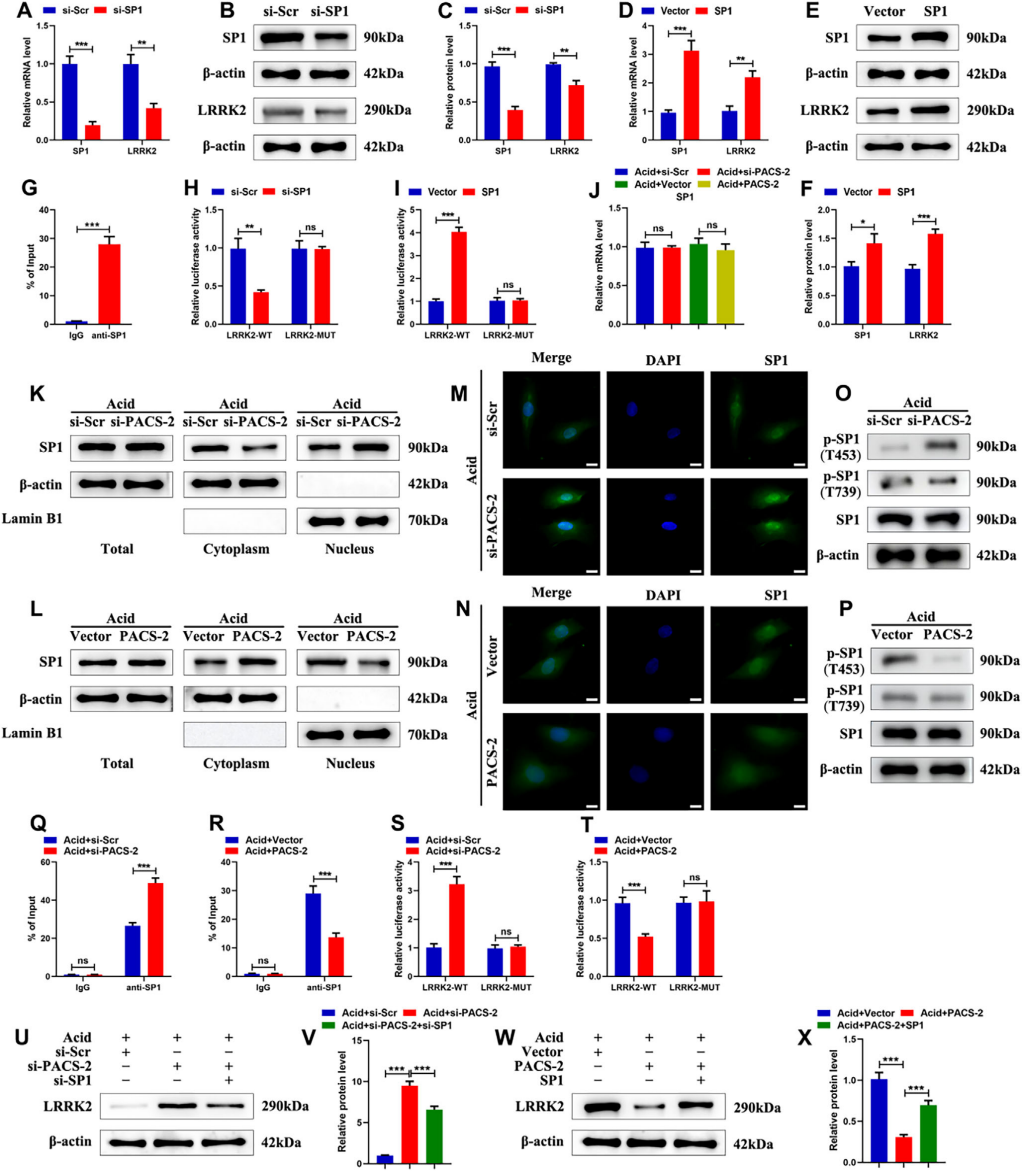
PACS‐2 inhibits the expression of LRRK2 via SP1. A) The relative mRNA levels of SP1 and LRRK2 in NPSCs treated with si‐Scr and si‐SP1 (n = 3). B,C) Representative western blot images and quantitative analysis of SP1 and LRRK2 in NPSCs treated with si‐Scr and si‐SP1 (n = 3). D) The relative mRNA levels of SP1 and LRRK2 in NPSCs treated with SP1 plasmid and control vector (n = 3). E,F) Representative western blot images and quantitative analysis of SP1 and LRRK2 in NPSCs treated with SP1 plasmid and control vector (n = 3). G) ChIP assays to show the enrichment of SP1 in the promoter region of LRRK2 in NPSCs (n = 3). H) Luciferase reporter assays were performed by co‐transfecting the WT LRRK2 promoter reporter vector or MUT LRRK2 promoter reporter vector with si‐Scr or si‐SP1 (n = 3). I) Luciferase reporter assays were performed by co‐transfecting the WT LRRK2 promoter reporter vector or MUT LRRK2 promoter reporter vector with SP1 plasmid or control vector (n = 3). J) The relative mRNA levels of SP1 in NPSCs treated with si‐PACS‐2, PACS‐2 plasmid, and their corresponding controls under acidic conditions (n = 3). K) Representative western blot images of SP1 in whole cell, cytoplasm, and nucleus of NPSCs treated with si‐Scr and si‐PACS‐2 (n = 3). L) Representative western blot images of SP1 in whole cell, cytoplasm, and nucleus of NPSCs treated with PACS‐2 plasmid and control vector (n = 3). M,N) Representative immunofluorescence staining images of SP1 in NPSCs treated with si‐PACS‐2, PACS‐2 plasmid, and their corresponding controls under acidic conditions. Scale bar: 10 µm. O) Representative western blot images of p‐SP1 (T453) and p‐SP1 (T739) in NPSCs treated with si‐Scr and si‐PACS‐2 (n = 3). P) Representative western blot images of p‐SP1 (T453) and p‐SP1 (T739) in NPSCs treated with PACS‐2 plasmid and control vector (n = 3). Q) ChIP assays to examine the effect of si‐PACS‐2 transfection on the binding of SP1 to LRRK2 promoter region in NPSCs (n = 3). R) ChIP assays to examine the effect of PACS‐2 plasmid transfection on the binding of SP1 to LRRK2 promoter region in NPSCs (n = 3). S) Luciferase reporter assays were performed by co‐transfecting the WT LRRK2 promoter reporter vector or MUT LRRK2 promoter reporter vector with si‐Scr or si‐PACS‐2 (n = 3). T) Luciferase reporter assays were performed by co‐transfecting the WT LRRK2 promoter reporter vector or MUT LRRK2 promoter reporter vector with PACS‐2 plasmid or control vector (n = 3). U,V) Representative western blot images and quantitative analysis of LRRK2 in NPSCs treated with si‐PACS‐2 or co‐treated with si‐PACS‐2 and si‐SP1 under acidic conditions (n = 3). W,X) Representative western blot images and quantitative analysis of LRRK2 in NPSCs treated with PACS‐2 plasmid or co‐treated with PACS‐2 plasmid and SP1 plasmid under acidic conditions (n = 3). Data are presented as mean ± SD. ns, not significant, ^*^ *p* < 0.05, ^**^ *p* < 0.01, and ^***^ *p* < 0.001.

To investigate the mechanism by which PACS‐2 regulates LRRK2 transcription in NPSCs, we focused on the role of the transcription factor SP1, which has been shown to control LRRK2 expression.^[^ ^20^ ^]^ SP1 knockdown reduced LRRK2 expression at both mRNA and protein levels in NPSCs (**Figure** **6**A–C), whereas SP1 overexpression increased LRRK2 expression (Figure 6D–F). Chromatin immunoprecipitation (ChIP) assays confirmed significant enrichment of SP1 at the LRRK2 promoter region (Figure 6G). Luciferase reporter assays further demonstrated that SP1 knockdown decreased, whereas SP1 overexpression increased, the luciferase activity of the WT LRRK2 promoter reporter. These changes were not observed with the mutant‐type (MUT) LRRK2 promoter reporter (Figure 6H,I), indicating that SP1 directly binds the LRRK2 promoter to regulate its transcription in NPSCs.

Next, we examined whether PACS‐2 regulates SP1 expression and nuclear localization in acid‐treated NPSCs. qRT‐PCR and western blot analysis indicated no significant change in the mRNA or total protein levels of SP1 following PACS‐2 knockdown or overexpression (Figure 6J–L). Notably, PACS‐2 knockdown increased nuclear SP1 and decreased cytoplasmic SP1 protein levels, whereas PACS‐2 overexpression showed the opposite effect (Figure 6K,L; Figure S5A,B, Supporting Information). Immunofluorescence staining confirmed that PACS‐2 overexpression resulted in cytoplasmic retention of SP1, whereas PACS‐2 knockdown promoted nuclear translocation of SP1 in acid‐treated NPSCs (Figure 6M,N). Moreover, SP1 phosphorylation has been reported to be closely related to its nuclear localization. While SP1 phosphorylation at Thr739 (T739) showed no significant change following PACS2 manipulation, phosphorylation at Thr453 (T453) was specifically observed to increase upon PACS‐2 knockdown and decrease upon its overexpression (Figure 6O,P; Figure S5C,D, Supporting Information). In addition, PACS‐2 knockdown increased SP1 enrichment at the LRRK2 promoter, whereas PACS‐2 overexpression reduced it (Figure 6Q,R). Consistently, upregulation of PACS‐2 decreased, and downregulation of PACS‐2 increased, the luciferase activity of the LRRK2‐WT reporter, with no significant effects on the LRRK2‐MUT reporter (Figure 6S,T). Furthermore, western blot analysis showed that PACS‐2 knockdown‐induced upregulation of LRRK2 was inhibited by co‐transfection with si‐SP1, whereas PACS‐2 overexpression‐induced downregulation of LRRK2 was reversed by co‐transfection with SP1 plasmid in NPSCs under acidic conditions (Figure 6U–X). These findings indicate that PACS‐2 represses LRRK2 transcription in NPSCs by promoting cytoplasmic retention of SP1 and thereby preventing its nuclear translocation.

### 2.6 Enhancing Pacs‐2 Expression in NPSCs Improves the Efficacy of Stem Cell Transplantation in a Rat Model of IDD

**Figure 7:**
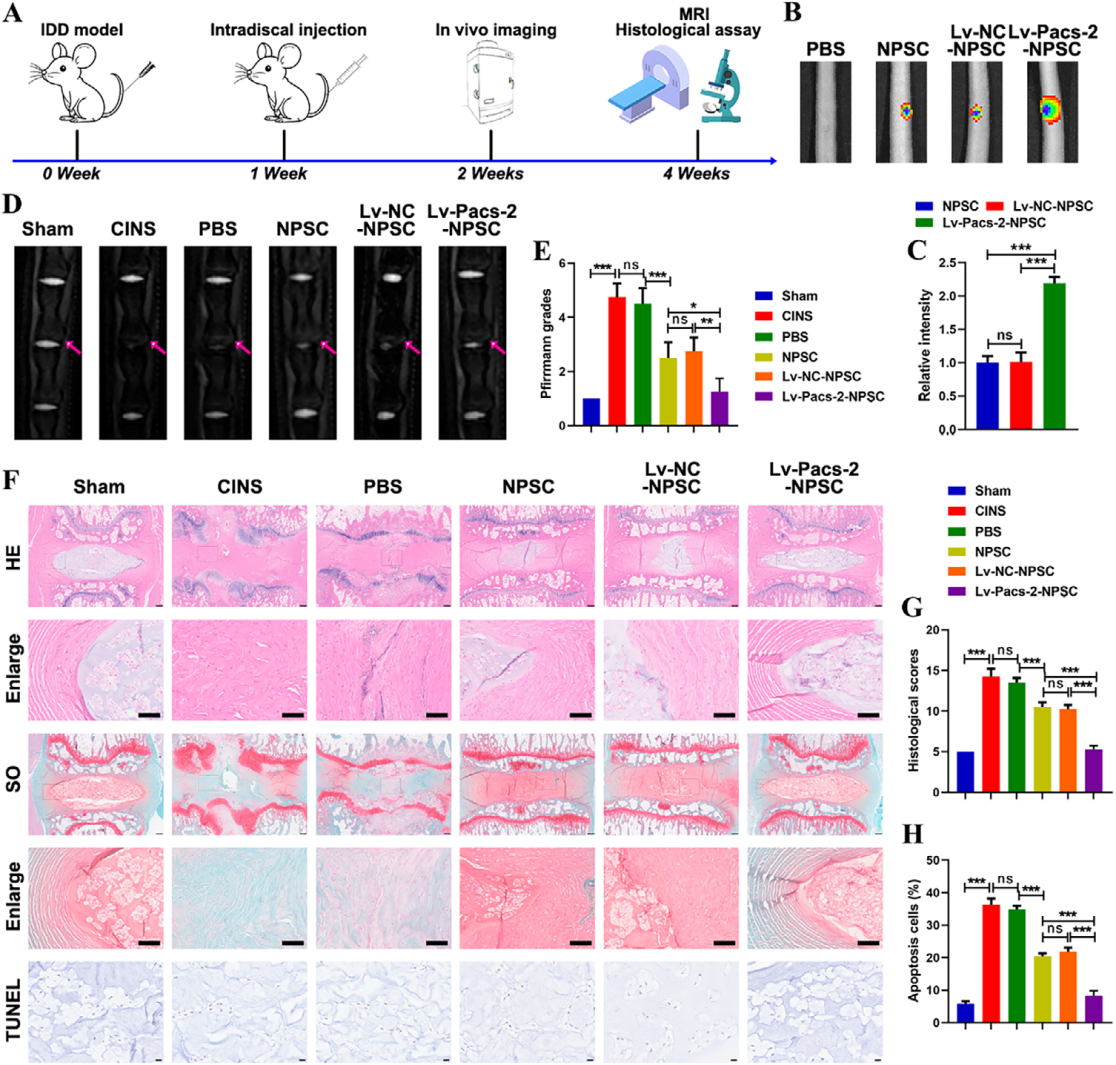
Pacs‐2‐overexpressing NPSCs inhibit IDD in vivo. A) Schematic diagram explaining the workflow of animal experiments in this study. B) Representative images showing the fluorescence intensity at one week after NPSC transplantation. C) Quantitative analysis of fluorescence intensity (n = 5). D) Representative MRI images of rat tails. E) The MRI Pfirrmann grade analysis of the rat tail IVDs (n = 5). F) Representative HE staining images (Scale bar: upper panel, 200 µm; lower panel, 100 µm), SO staining images (Scale bar: upper panel, 200 µm; lower panel, 100 µm), and TUNEL staining images (Scale bar: 10 µm) of rat IVD tissues. G) Histological scores of rat IVD tissues (n = 5). H) The proportion of apoptotic cells calculated as the percentage of TUNEL‐positive cells (n = 5). Data are presented as mean ± SD. ns, not significant, ^*^ *p* < 0.05, ^**^ *p* < 0.01, and ^***^ *p* < 0.001.

Based on these findings, we hypothesized that NPSCs overexpressing PACS‐2 would exhibit greater resistance to the deleterious microenvironment following transplantation into degenerated IVDs, thereby providing enhanced efficacy in delaying IDD progression. To test this, rat NPSCs were transduced with Pacs‐2 using a lentiviral vector (Lv), and successful overexpression was confirmed (Figure S6A–C, Supporting Information). These cells were labeled with DiR and transplanted into the operated disc segments of a rat IDD model. The workflow is shown in **Figure** **7**A. In vivo imaging, HE staining, SO staining, TUNEL staining, and MRI were utilized to evaluate the therapeutic effect of intra‐disc transplantation of NPSCs.

One week post‐transplantation, in vivo imaging revealed that the fluorescence intensity of DiR‐labeled NPSCs in the Lv‐Pacs‐2‐NPSC group was higher than that in the Lv‐negative control (NC)‐NPSC and NPSC groups, indicating improved cell survival (Figure 7B,C). Four weeks after surgery, MRI showed that the T2‐weighted signal intensity in the NPSC group was greater than that in the PBS group, and the Lv‐Pacs‐2‐NPSC group had the highest signal intensity than that in the Lv‐NC‐NPSC group and the NPSC group (Figure 7D). Pfirrmann grading further supported that transplantation with Lv‐Pacs‐2‐NPSCs more effectively reversed the progression of IDD (Figure 7E). HE and SO staining of IVD specimens collected at four weeks post‐operation demonstrated that NPSC transplantation mitigated puncture‐induced degenerative changes, such as NP atrophy and disordered IVD architecture (Figure 7F). The Lv‐Pacs‐2‐NPSC group exhibited a more intact NP and a clearer boundary between the NP and annulus fibrosus than the NPSC and Lv‐NC‐NPSC groups (Figure 7F). Histological scoring showed that the PBS group had higher scores than the NPSC group, and the Lv‐Pacs‐2‐NPSC group had lower scores than both the NPSC and Lv‐NC‐NPSC groups (Figure 7G). TUNEL staining revealed that the Lv‐Pacs‐2‐NPSC group had a lower apoptosis level than both the NPSC and Lv‐NC‐NPSC groups (Figure 7F,H). These data indicate that PACS‐2 enhances NPSC survival following transplantation and supports its therapeutic potential in treating IDD.

## 3 Discussion

**Figure 8:**
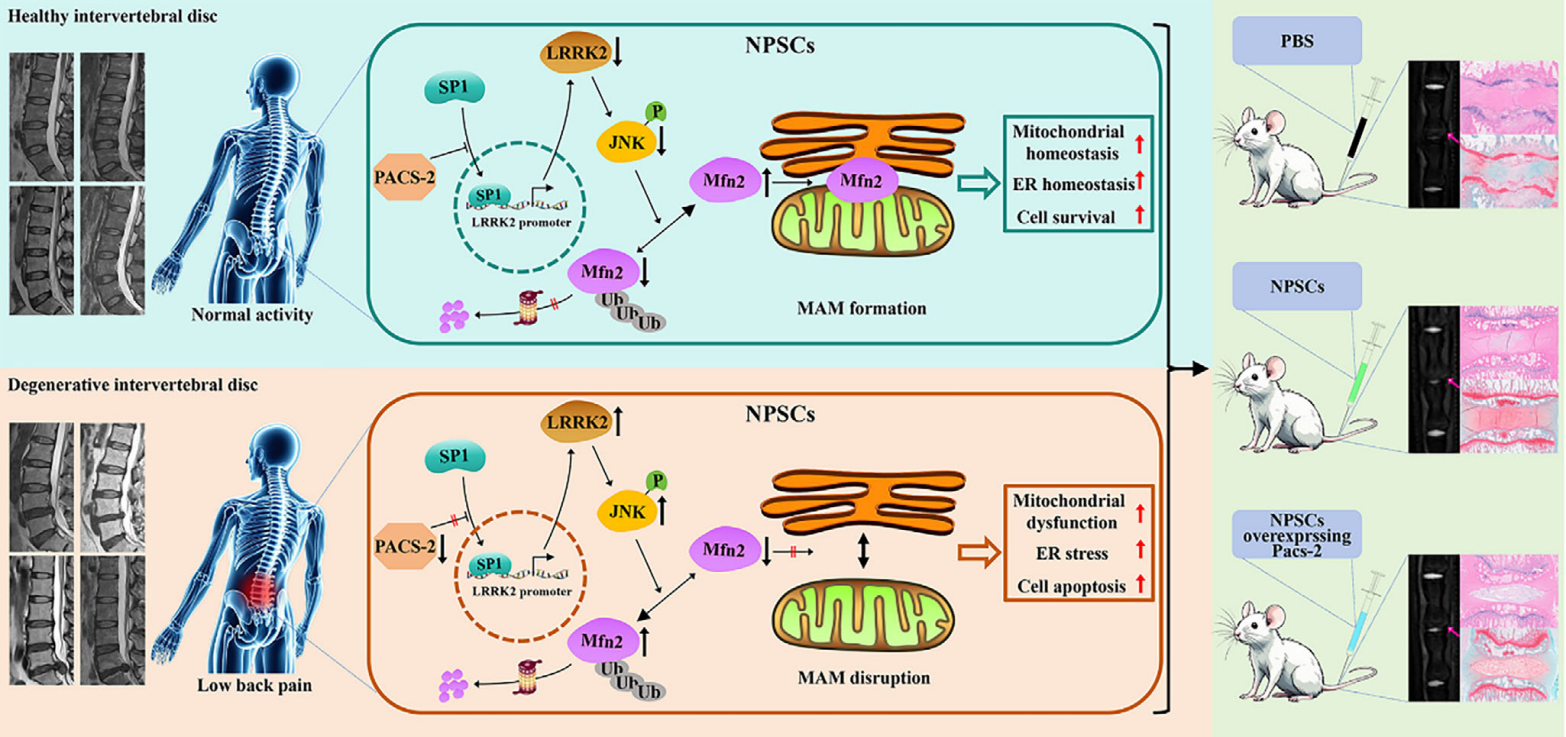
Schematic diagram illustrating the molecular mechanism of PACS‐2 in regulating IVD homeostasis and the strategy of retarding IDD based on NPSC transplantation. During the process of IDD, MAM integrity is significantly impaired, accompanied by decreased PACS‐2 expression and increased apoptosis levels. Mechanistically, PACS‐2 suppresses the nuclear translocation of SP1, thereby downregulating the expression of its downstream target LRRK2. This reduces LRRK2‐mediated ubiquitination and degradation of Mfn2 through the JNK pathway, preserving MAM integrity and promoting NPSC survival. In vivo, transplantation of Pacs‐2–overexpressing NPSCs improved cell survival and enhanced IVD repair in a rat model.

Currently, the clinical treatments for IDD primarily include physical therapy, medication, and surgical intervention. These approaches are aimed solely at alleviating pain symptoms and cannot halt or reverse the progressive development of IDD. This limitation often leads to recurrent and worsening pain symptoms in patients. With an increasingly aging population and changing lifestyles, the demand for effective therapies has become more urgent. Stem cell‐based therapies have shown promising performance in various diseases, and their effectiveness in treating IDD has been reported in multiple studies.^[^ ^21^, ^22^, ^23^ ^]^ However, the harsh acidic microenvironment in degenerative IVDs, characterized by nutrient deficiency and metabolic waste accumulation, significantly impairs repair and regeneration mediated by both endogenous and transplanted stem cells, a challenge that remains unresolved.^[^ ^24^ ^]^ In this study, we demonstrated that PACS‐2‐mediated MAM integrity is compromised during the progression of IDD, and that PACS‐2 knockout accelerates IDD. Mechanistically, PACS‐2 maintains MAM integrity through the SP1/LRRK2/Mfn2 pathway, thereby inhibiting acid‐induced apoptosis of NPSCs. Transplantation of Pacs‐2‐overexpressing NPSCs effectively prevented the progression of IDD. These results highlight the essential role of PACS‐2 in maintaining IVD homeostasis and highlight its effect on NPSC‐based IVD repair and regeneration (**Figure** **8**).

Mitochondria and the ER are key organelles that participate in many pathophysiological processes, and their roles in IDD are well established.^[^ ^25^, ^26^ ^]^ Coordinated mitochondrial dynamics and mitophagy can target and degrade damaged mitochondria, thereby restoring mitochondrial homeostasis and preventing IDD.^[^ ^27^ ^]^ Loss of IVD cells and disruption of the matrix anabolism–catabolism balance, as caused by ER stress, accelerate IDD progression.^[^ ^28^ ^]^ Mitochondria and ER are now recognized as interconnected structures through the MAM, which serves as a scaffold for their crosstalk and is critical for cellular health.^[^ ^29^, ^30^, ^31^ ^]^ For instance, Ca^2+^ signal transmission between the ER and mitochondria is a prominent function of the MAM. After being released from the ER, Ca^2+^ enters mitochondria via MAM protein channels. Normal mitochondrial Ca^2+^ uptake increases tricarboxylic acid cycle and complex activity, promoting ATP production, but excessive Ca^2+^ leads to mitochondrial permeability transition pore opening and induces apoptosis.^[^ ^12^, ^32^ ^]^ Preliminary studies revealed that disruption of MAM structure is present in degenerative NPCs, and that enhancing MAM coupling promotes NPC proliferation.^[^ ^33^ ^]^

The levels of several anchor proteins in the MAM have been reported to regulate its function by altering the ER–mitochondria distance, affecting cellular homeostasis.^[^ ^34^, ^35^ ^]^ The role of PACS‐2 in maintaining MAM structure and function has been established.^[^ ^13^ ^]^ Notably, PACS‐2‐mediated MAM homeostasis displays different effects under various stimuli and in different disease models, likely related to the degree of ER–mitochondrial coupling and its effect on signal transduction.^[^ ^14^, ^15^ ^]^ However, the importance of PACS‐2‐mediated MAM homeostasis in various diseases is widely recognized. In this study, we found that PACS‐2 expression was decreased and MAM integrity was disrupted during IDD, accompanied by increased apoptosis. Compared to IDD mice, IDD Pacs‐2^–/–^ mice exhibited more severe degeneration. Importantly, PACS‐2 overexpression in NPSCs restored acid‐induced MAM disruption and reduced apoptosis. Co‐overexpression of FATE‐1 significantly attenuated the protective effects induced by PACS‐2, confirming that the effect of PACS‐2 is associated with its regulation of MAM function.

To further investigate the mechanism by which PACS‐2 maintains MAM integrity, we performed transcriptomic analysis and found that PACS‐2 knockdown significantly increased LRRK2 expression in NPSCs. Elevated LRRK2 levels were also observed in degenerative NP tissues and acid‐treated NPSCs. LRRK2, a kinase best known for its role in Parkinson's disease, is also associated with inflammatory responses, autophagy, and mitochondrial homeostasis.^[^ ^36^, ^37^, ^38^ ^]^ Most research on LRRK2 focuses on the nervous system, but its roles elsewhere are rarely studied. Notably, LRRK2 can induce the loss of Mfn2 through ubiquitin‐proteasome–mediated degradation.^[^ ^19^ ^]^ Mfn2 was initially identified as a mitochondrial outer membrane protein that participates in mitochondrial fusion and was subsequently recognized as important for endoplasmic reticulum–mitochondria contact.^[^ ^39^, ^40^ ^]^ It connects the ER and mitochondria through interactions between Mfn2 on the ER and Mfn2 or Mfn1 on mitochondria.^[^ ^41^, ^42^ ^]^ Although some studies suggest Mfn2 is a negative regulator of MAM formation,^[^ ^43^, ^44^ ^]^ our results indicate that Mfn2 knockdown reduces MAM formation, suggesting a positive role for Mfn2 in MAMs in NPSCs. Previous studies have confirmed that under cellular stress, JNK induces the phosphorylation of Mfn2 at Ser27, which recruits the HECT domain E3 ubiquitin ligase Huwe1, subsequently leading to the ubiquitination and degradation of Mfn2.^[^ ^17^ ^]^ As a member of the MLK subfamily of MAPKKK, LRRK2 activates the JNK pathway.^[^ ^18^ ^]^ Our data show that LRRK2 promotes the ubiquitination and degradation of Mfn2, accompanied by increased phosphorylation JNK and Mfn2. Inhibition of JNK reverses LRRK2‐induced Mfn2 phosphorylation and degradation. Functional experiments demonstrated that PACS‐2 reduces Mfn2 degradation through the LRRK2/JNK pathway, thereby maintaining MAM integrity and NPSC homeostasis. Therefore, these results indicate that during IDD, the decreased expression level of PACS‐2 in NPSCs leads to the degradation of Mfn2, thereby disrupting the integrity of MAM. This results in impaired MAM function, such as obstruction of Ca^2+^ transfer between ER/mitochondria, leading to mitochondrial dysfunction and ER stress, ultimately promoting NPSC apoptosis.

We further explored how PACS‐2 regulates LRRK2 expression. SP1 is a transcription factor that binds GC‐rich promoter sequences and plays significant roles in growth, differentiation, apoptosis, inflammation, and oxidative stress.^[^ ^45^, ^46^, ^47^ ^]^ SP1 expression is elevated during IDD, and its inhibition slows IDD progression.^[^ ^48^ ^]^ A regulatory association between SP1 and LRRK2 has been confirmed.^[^ ^20^ ^]^ We found that SP1 directly binds the LRRK2 promoter to regulate its expression in NPSCs. PACS‐2 does not affect total SP1 protein levels but inhibits its nuclear translocation. The activity of SP1 has been revealed to be strictly regulated by its phosphorylation and acetylation modifications.^[^ ^49^, ^50^, ^51^, ^52^ ^]^ Among these, the phosphorylation of SP1 at T453 and T739 is associated with its nuclear localization.^[^ ^52^ ^]^ Our results indicated that the phosphorylation of SP1 at T453, but not T739, may mediate the PACS‐2‐regulated nuclear translocation of SP1 in NPSCs. PACS‐2 is a membrane transporter protein. Its unique structure enables it to bind to cargo proteins and transport them to corresponding subcellular structures. Yang et al. demonstrated that PACS‐2 can bind to the transcription factor EB and regulate its activity.^[^ ^53^ ^]^ Therefore, we speculate that PACS‐2 may regulate the phosphorylation level of SP1 by recruiting specific phosphokinases or phosphatases. The specific molecular mechanisms involved still need to be further explored in the future. These results indicate that PACS‐2 suppresses LRRK2 expression by preventing SP1 nuclear translocation.

Based on these findings regarding the role of PACS‐2 in IDD development and in acid‐treated NPSCs, we propose that NPSCs overexpressing PACS‐2 are more capable of resisting the harsh microenvironment in degenerative IVDs. We generated Pacs‐2‐overexpressing NPSCs via lentiviral transfection and assessed whether this approach could improve the therapeutic efficacy of NPSC transplantation in an IDD model. As expected, these pretreated NPSCs demonstrated greater resistance to the degenerative microenvironment, with recipient IVDs displaying higher water content. Histological analysis further confirmed this protective effect. These results suggest that PACS‐2 has substantial potential to enhance the efficacy of NPSC transplantation.

However, several limitations remain. First, although these results demonstrate that the PACS‐2/SP1/LRRK2/Mfn2 axis inhibits NPSC apoptosis by maintaining MAM integrity, its clinical therapeutic value still requires further validation through expanding human sample sizes and future preclinical safety and efficacy assessments. Second, although numerous studies on IDD mechanisms have used animal models in rats and mice, including acupuncture, compression, and lumbar spine instability models, these models do not fully replicate the entire pathological process of IDD in humans.^[^ ^54^, ^55^, ^56^ ^]^ Future research using primate models may provide more relevant insights. Third, compared with IVD‐specific conditional PACS‐2 knockout mice, the effect of global PACS‐2 knockout introduces complex phenotypes that should be considered. Fourthly, although we have provided new insights into the molecular mechanisms by which PACS‐2 regulates MAM integrity, particularly through the SP1/LRRK2/Mfn2 pathway, our RNA sequencing results also identified many other molecules and pathways whose roles in PACS‐2–regulated MAM integrity remain to be determined. Finally, the pathological mechanism of IDD is very complex, which is currently the main reason for its poor clinical treatment effectiveness. Although this study proposes a new mechanism for IDD, its correlation with other signaling pathways has not been fully elucidated. For example, ER stress is a potent activator of classic inflammatory pathways such as NF‐κB.^[^ ^57^ ^]^ The dysfunction of MAM and ER stress caused by PACS‐2 deficiency may be one of the upstream driving factors of inflammatory response in IDD. In addition, ER stress and mitochondrial dysfunction can jointly regulate the metabolic balance of extracellular matrix through signal transduction and energy supply, leading to a tendency for degradation activity to be higher than synthesis activity, ultimately resulting in the destruction of extracellular matrix.^[^ ^58^ ^]^ Therefore, further research is needed in the future to verify the relationship between these IDD‐related pathways.

## 4 Conclusion

In summary, this study demonstrates the contribution of PACS‐2 to IDD progression and its protective effect against acid‐induced apoptosis of NPSCs. PACS‐2 inhibits the nuclear translocation of SP1, thereby reducing LRRK2 expression and subsequent JNK‐mediated ubiquitination and degradation of Mfn2. This process promotes MAM formation and enhances NPSC survival. These findings highlight the beneficial role of PACS‐2 in intrinsic IVD repair and stem cell–based IVD regeneration, presenting PACS‐2 as a promising therapeutic target for IDD treatment.

## 5 Experimental Section

### Human NP Tissue Samples

Human NP tissue samples were collected from sixteen patients undergoing spinal surgery for lumbar disc herniation or lumbar fracture. The degenerative grade of each tissue sample was evaluated using the MRI‐based Pfirrmann grading system.^[^ ^59^ ^]^ All patient's information is shown in Table S1 (Supporting Information). This study received approval from the Ethics Committee of the First Affiliated Hospital of Anhui Medical University (approval no. PJ20230729), and all participants provided informed consent.

### Animal Experiments

All animal experiments were approved by the Animal Ethics Committee of Anhui Medical University (approval no. LLSC20220096). A CINS model was established in Sprague–Dawley rats (Experimental Animal Center of Anhui Medical University) and Pacs‐2^−/−^ mice (Shanghai Model Organisms Center), following previously described procedure.^[^ ^1^, ^8^ ^]^

Thirty SD rats were randomly assigned to six groups (n = 5 per group):

- Lv‐Pacs‐2‐NPSC group: NPSCs overexpressing Pacs‐2, labeled with 1,1‐dioctadecyl‐3,3,3,3‐tetramethylindotricarbocyanine iodide (DiR; AAT Bioquest, CA, USA), were transplanted into the operated disc segments.
- Lv‐NC‐NPSC group: NPSCs transfected with NC lentivirus, labeled with DiR, were transplanted into the same disc.
- NPSC group: Untransfected NPSCs, labeled with DiR, were transplanted into the same disc.
- PBS group: The same volume of phosphate‐buffered saline was injected.
- CINS group: Puncture operation was performed without injection.
- Sham group: The same needle puncture as in the CINS group was performed, but only the corresponding skin area was punctured, leaving the IVD intact.

Fluorescence intensity of DiR‐labeled NPSCs was detected using an in vivo imaging system (IVIS Lumina III, PerkinElmer, CA, USA) one week after intra‐disc transplantation. Four weeks post‐surgery, all rats underwent MRI, and the progression of IDD was evaluated using the Pfirrmann grading system. The rats were then euthanized and IVDs were collected for further analysis.

Pacs‐2^−/−^ mice were obtained on a C57BL/6 background. WT and Pacs‐2^−/−^ mice were randomly assigned to Sham or CINS groups (n = 5 per group). Four weeks after surgery, mouse IVDs were analyzed by imaging and histology.

### Histology and Immunostaining Assays

Human NP tissue sections fixed in paraformaldehyde and embedded in paraffin were stained with HE and Alcian Blue. For IVD tissues from rats and mice, paraffin‐embedded tissue sections were stained with HE and SO. Histological scoring was performed according to established IVD histological criteri.^[^ ^60^ ^]^ Immunohistochemistry and immunofluorescence staining for specific proteins and MAM integrity were performed using the appropriate primary antibodies. TUNEL staining was conducted on NP tissue sections using a TUNEL assay kit (Roche, Switzerland) according to the manufacturer's instructions. Images were captured by a fluorescence microscope (Leica, Germany). Antibody details are provided in Table S2 (Supporting Information).

### Analysis of Single‐Cell RNA Sequencing (ScRNA‐seq) Data of Human NP Tissues

The scRNA‐seq dataset and clinical information were obtained from the Gene Expression Omnibus (GEO; GSE165722), which included a total of eight NP tissue samples with various degrees of IDD (six from lumbar disc herniation and two from burst fracture). The data of all patients were standardized in this dataset using the R package and analyzed the differences in NPCs with different degrees of IDD. Based on the Pfirrmann grading of the samples, it was classified into two categories: Pfirrmann II/III and Pfirrmann IV/V NP tissues. According to previous research results, CD90+ NPCs in fibroNPCs exhibits stem cell characteristics (PMID: 34825784).^[^ ^16^ ^]^ Fibrosis related genes (COL1A1, COL3A1, and COL6A1) were used along with the CD90 gene as cell identification markers. Subsequently, based on PACS‐2 gene expression, the CD90+NPCs population was divided into CD90+/PACS‐2+ NPCs and other cell populations. Finally, the proportions of CD90+/PACS‐2+ NPCs in Pfirrmann II/III and Pfirrmann IV/V NP tissues were analyzed and compared.

### Isolation and Culture of NPSCs

Primary NPSCs were isolated from NP tissues of SD rats and humans using the previously described method.^[^ ^7^, ^9^ ^]^ Specifically, NP tissues isolated from peripheral annulus fibrous tissues were washed three times with sterile PBS, carefully cut into small pieces with sterile ophthalmic scissors, and enzymatically digested with 0.2% type II collagenase (Gibco, USA) at 37 °C for 4 h. After the tissue suspension was centrifuged at 1000×g for 5 min, the collected cells were resuspended and cultured in low‐glucose Dulbecco's modified Eagle's medium (HyClone, USA) containing 10% fetal bovine serum (Gibco, USA) and 1% penicillin–streptomycin, at 37 °C with 5% carbon dioxide. The culture medium was replaced twice weekly. Second‐generation cells were used for subsequent experiments. To establish an acidic microenvironment, the medium was adjusted to pH 6.5 by adding sterile HCl, with pH confirmed using a commercial pH microelectrode (Lazarlab).

### Cell Transfection

siRNA targeting human PACS‐2, LRRK2, Mfn2, SP1, and scrambled control siRNA (si‐Scr) were synthesized by Corues Biotechnology (Nanjing, China). siRNA sequences are provided in Table S3 (Supporting Information). Plasmids for overexpressing human PACS‐2, FATE‐1, LRRK2, SP1, and a negative control were synthesized by Applied Biological Materials. Transfections were performed using Lipofectamine 3000 (Invitrogen, CA) according to the manufacturer's protocol. For lentiviral infection, rat NPSCs were transduced with rat Pacs‐2‐expressing or control lentivirus (Lv‐Pacs‐2 or Lv‐NC, Applied Biological Materials) at a multiplicity of infection (MOI) of 100. Transfection efficiency was evaluated by qRT‐PCR and Western blot prior to transplantation.

### Characterization of NPSCs

NPSCs were washed, resuspended in PBS, and incubated with antibodies for CD105, CD73, CD90, CD34, and CD45 in accordance with ISCT criteria for stem cell identification. Labeled NPSCs were analyzed by flow cytometry (Beckman, USA) following standard protocols. To assess multilineage differentiation potential, osteogenic, adipogenic, and chondrogenic differentiation was induced using commercial kits. Alizarin Red (Procell, Wuhan, China), Oil Red O (Procell, Wuhan, China), and Alcian Blue (Procell, Wuhan, China) staining were performed as instructed by the manufacturer.

### Assessment of the MAM Integrity In Vitro

MAM integrity was evaluated by immunofluorescence as described previously.^[^ ^61^ ^]^ NPSCs were stained with Mito‐Tracker and ER‐Tracker dyes (Beyotime, China), and nuclei were counterstained with DAPI. Images were acquired using a laser scanning confocal microscope (ZEISS, Germany).

ER–mitochondria contacts were observed by transmission electron microscopy (TEM). The length of the mitochondrial outer membrane associated with the ER and the mitochondrial perimeter was measured using ImageJ software. MAM integrity was calculated according to established protocols.^[^ ^61^ ^]^

### Cell Immunofluorescence Staining

Cell immunofluorescence staining was performed as previously described.^[^ ^9^ ^]^ NPSCs cultured on slides were fixed with 4% paraformaldehyde and permeabilized with 0.1% Triton X‐100 (Biosharp). After PBS washing, cells were blocked with 2% bovine serum albumin (BSA), incubated with primary antibodies at 4 °C overnight, and then with appropriate secondary antibodies. After DAPI (Beyotime, China) counterstaining, images were captured using a fluorescence microscope (Leica, Germany). Antibody details are provided in Table S2 (Supporting Information).

### Western Blot Analysis and Co‐Immunoprecipitation (Co‐IP)

Total protein was extracted from NP tissues or NPSCs using RIPA lysis buffer (Beyotime, China). Nuclear and cytoplasmic proteins were extracted using a Nuclear and Cytoplasmic Protein Extraction Kit (Beyotime, China). Protein samples were separated by SDS‐PAGE and transferred onto PVDF membranes (Bio‐Rad, Hercules, CA, USA). Membranes were incubated with primary and secondary antibodies and visualized using an enhanced chemiluminescence kit. Band intensity was quantified using ImageJ software. Antibody information is listed in Table S2 (Supporting Information).

For Co‐IP, anti‐Mfn2 or control IgG antibodies were used, followed by incubation with Protein A/G PLUS‐Agarose (Santa Cruz Biotechnology, CA, USA). The agarose–antibody–protein complex was collected and analyzed by western blot.

### Detection of Cell Apoptosis

Apoptosis rates of NPSCs were determined using the Annexin V‐FITC/PI apoptosis kit (Bestbio, China), following the manufacturer's instructions. Cells were collected, resuspended in binding buffer, incubated with Annexin V‐FITC for 20 min and PI for 5 min at 4 °C in the dark, and analyzed by flow cytometry (Beckman, USA).

### Quantitative Real‐Time PCR (qRT‐PCR)

Total RNA was extracted from NPSCs using TRIzol reagent (Invitrogen, CA, USA). Reverse transcription and amplification were performed according to the manufacturer's protocol (Biosharp, China). Primer sequences are listed in Table S4 (Supporting Information). Cycle threshold (Ct) values were normalized to β‐actin. Gene expression was calculated using the 2^–ΔΔCt^ method.

### Measurement of Mitochondrial Membrane Potential (MMP), Intracellular ROS Production, and ATP Content

MMP, intracellular ROS production, and ATP content were measured using commercial reagent kits as described previously.^[^ ^10^, ^62^ ^]^ MMP was assessed using the JC‐1 probe (Beyotime, China). Intracellular ROS levels were measured using the DCFH‐DA fluorescent indicator (Beyotime, China). ATP content was determined using the ATP Assay Kit according to the manufacturer's instructions (Beyotime, China).

### RNA Sequencing (RNA‐seq)

Total RNA from NPSCs transfected with si‐PACS‐2 or si‐Scr under acidic conditions was extracted using TRIzol reagent (Invitrogen, CA, USA). RNA quality control, library construction, sequencing, and data analysis were performed by OE Biotech (Shanghai, China).

### Chromatin Immunoprecipitation (ChIP) Assay

ChIP assays to verify SP1 binding to the LRRK2 promoter were conducted using the EZ‐ChIP Chromatin Immunoprecipitation Kit (Millipore, Germany) following the manufacturer's instructions. The anti‐SP1 antibody was used for immunoprecipitation, with control IgG as a negative control. Enriched DNA fragments were subjected to qRT‐PCR.

### Luciferase Reporter Assay

Luciferase reporter assays were performed as previously described. To assess transcriptional regulation of LRRK2 by SP1, NPSCs were transfected with either WT or MUT LRRK2 promoter reporter vectors and co‐transfected with si‐SP1 or SP1 plasmid. Luciferase activity was measured using the Dual‐Luciferase Reporter Assay Kit (Promega).

### Statistical Analysis

Data are presented as mean ± standard deviation (SD) from at least three independent experiments. Statistical comparisons between two groups were made using the unpaired Student's t test. Comparisons among multiple groups were performed using one‐way analysis of variance (ANOVA) followed by Tukey's post hoc test. Analyses were conducted with GraphPad Prism 9.0 software. Statistical significance was defined as P < 0.05.

## Conflict of Interest

The authors declare no conflict of interest.

## Supporting information



Supporting Information

## Data Availability

The data that support the findings of this study are available from the corresponding author upon reasonable request.
