# Supplementary material for: PACS‐2 Mitigates NPSC Apoptosis and Intervertebral Disc Degeneration by Preserving MAM Integrity via the SP1/LRRK2/Mfn2 Axis
Source: Adv Sci (Weinh). 2025 Nov 5;13(4):e11781. doi: 10.1002/advs.202511781 (PMC12822392; doi:10.1002/advs.202511781)
Supplement: Supplementary file 1 — Supporting Information [file ADVS-13-e11781-s001.docx]

**Supporting Information**

**PACS-2 Mitigates NPSC Apoptosis And Intervertebral Disc Degeneration By Preserving MAM Integrity Via The SP1/LRRK2/Mfn2 Axis**

*Liang Kang^1,2,3^#, Jiaqi Wang^1,2,3^#, Chenhao Zhao^1,2,3^#, Qiuwei Li^1,2,3^#, Zhigang Zhang^1,2,3^, Huaqing Zhang^1,2,3^, Chongyu Jia^1,2,3^, Luping Zhou^1,2,3^, Yanxin Wang^1,2,3^, Yu Chen^1,2,3^, Kaixuan Li^1,2,3^, Xu Yan^1,2,3^, Jie Fang^4^, Haibao Wang^4^, Dandan Wang^5^, Pingping Su^5^, Jingyu Zhang^6^, Zhiwei Chen^6^, Renjie Zhang^1,2,3^*, Cailiang Shen^1,2,3^**

1 Department of Orthopedics and Spine Surgery, The First Affiliated Hospital of Anhui Medical University, Hefei 230022, China.

2 Laboratory of Spinal and Spinal Cord Injury Regeneration and Repair, The First Affiliated Hospital of Anhui Medical University, Hefei 230022, China.

3 Anhui Province Research Center for The Clinical Application of Digital Medical Technology, The First Affiliated Hospital of Anhui Medical University, Hefei 230022, China.

4 Department of Radiology, The First Affiliated Hospital of Anhui Medical University, Hefei 230022, China

5 Department of Neurology, The First Affiliated Hospital of Anhui Medical University, Hefei 230022, China

6 Laboratory Animal Core of Institute of Health and Medicine, Hefei Comprehensive National Science Center, Hefei 230031, China

* Correspondence to:

Department of Orthopedics and Spine Surgery, The First Affiliated Hospital of Anhui Medical University, 218 Jixi Road, Hefei 230022, China. E-mail: ayfy_scl1616@163.com (Cailiang Shen);

Department of Orthopedics and Spine Surgery, The First Affiliated Hospital of Anhui Medical University, 218 Jixi Road, Hefei 230022, China. E-mail: zhangrenjie1089@126.com (Renjie Zhang)

#Liang Kang, Jiaqi Wang, Chenhao Zhao, and Qiuwei Li contributed equally to this work and should be regarded as co-first authors.

**This file includes:**

**Supplementary Figures S1-S6**

**Supplementary Tables S1-S4**

**Supplementary Figures**


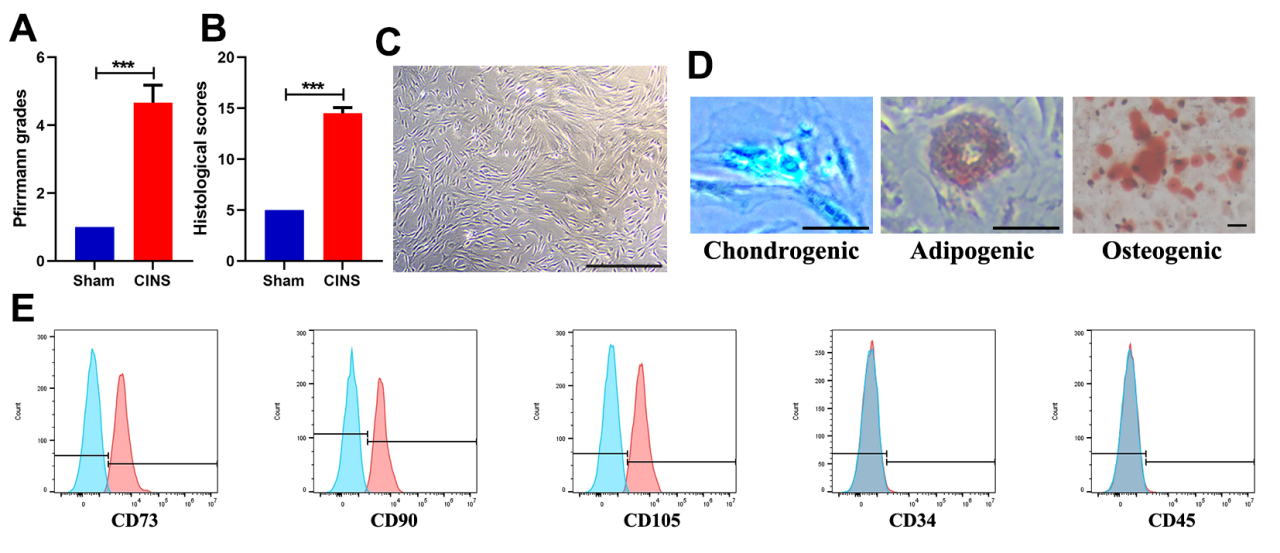


**Figure S1.** (A) The MRI Pfirrmann grade analysis of the rat tail IVDs (n=5). (B) Histological scores of rat IVD tissues (n=5). (C) Representative image showing the morphology of NPSCs. Scale bar: 250 μm. (D) NPSCs show positive staining for Alcian Blue, Oil Red O, and Alizarin Red after multilineage differentiation. Scale bar: 50 µm. (E) Flow cytometry analysis showing the surface marker of NPSCs. Data are presented as mean ± SD. ****p* < 0.001.


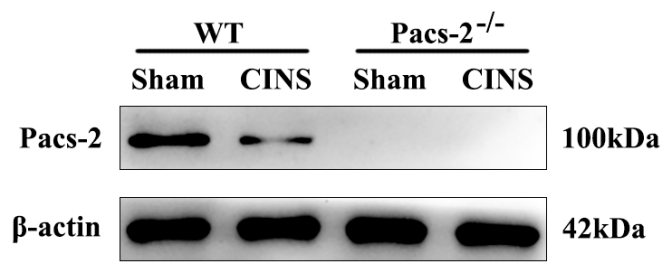


**Figure S2.** (A) Representative western blot images of Pacs-2 in Pacs-2 gene knockout mice and wild type mice.


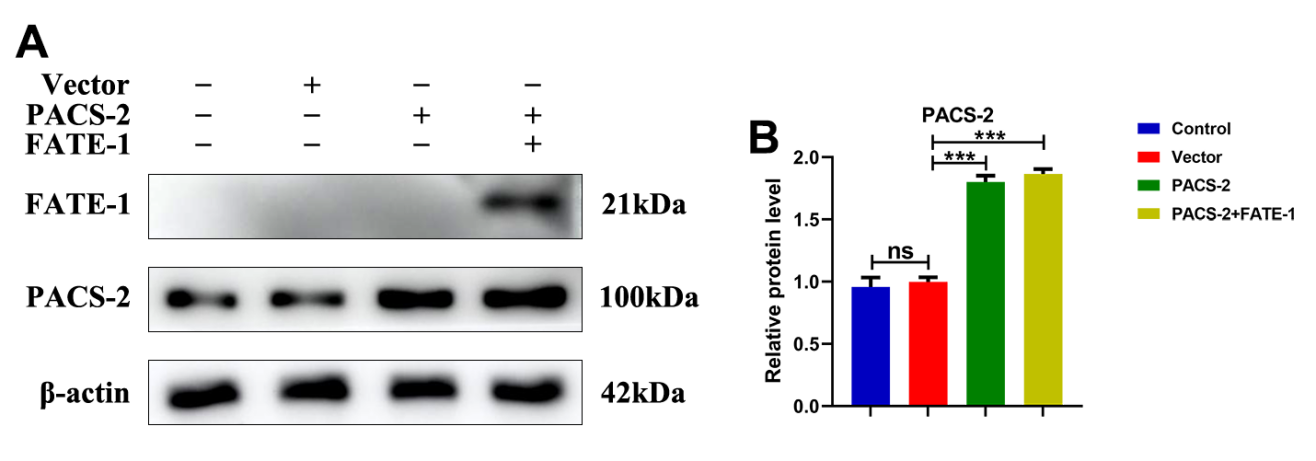


**Figure S3.** (A) Representative western blot images of FATE-1 and PACS-2 in NPSCs treated with PACS-2 plasmid or co-treated with PACS-2 plasmid and FATE-1 plasmid. (B) Quantitative analysis of the PACS-2 protein expression levels in (A) (n=3). Data are presented as mean ± SD. ns, not significant, ****p* < 0.001.


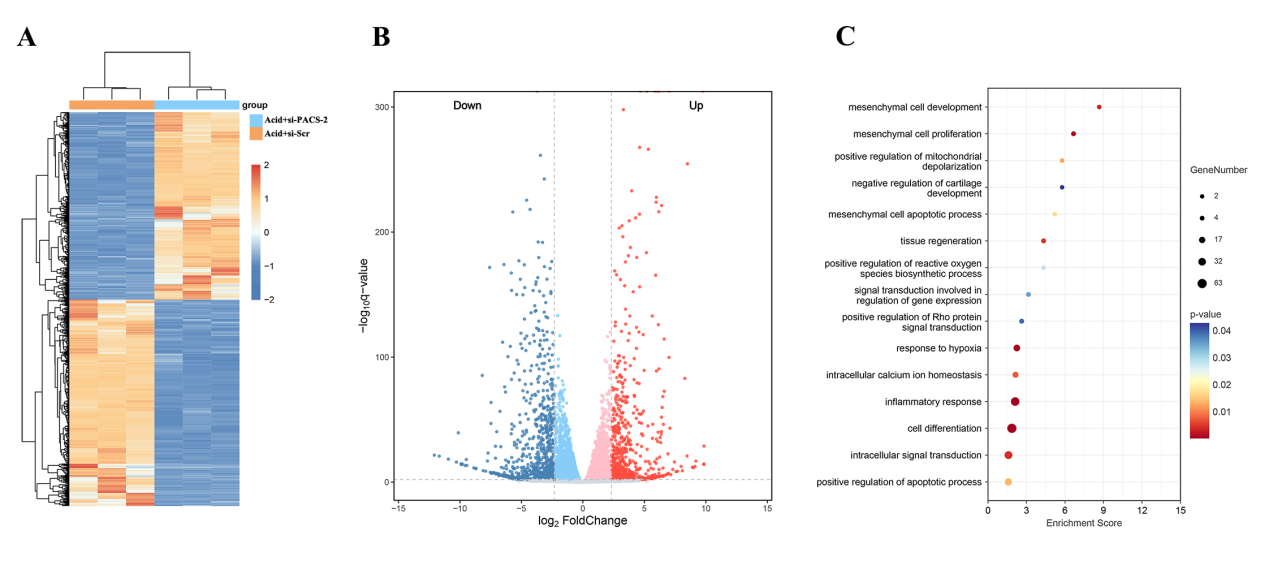


**Figure S4.** (A,B) Heatmap and volcano plot of differentially expressed genes (q-value < 0.01, |log2fold change| > 2.32) between Acid+si-Scr group and Acid+si-PACS-2 group. (C) Bubble chart of GO enrichment analysis (biological processes) of differentially expressed genes.


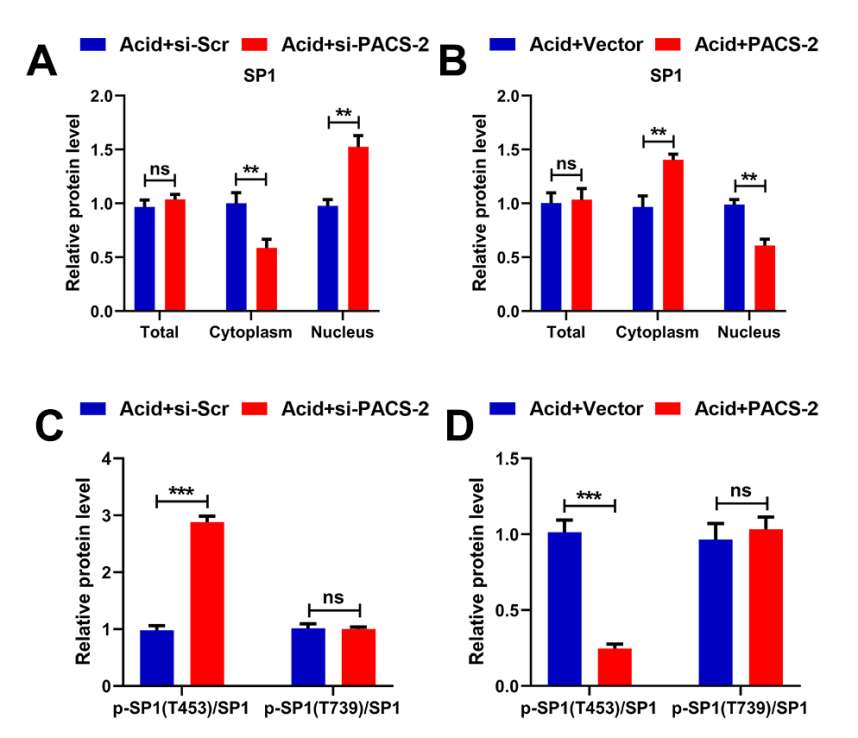


**Figure S5.** (A) Quantitative analysis of SP1 in whole cell, cytoplasm, and nucleus of NPSCs treated with si-Scr and si-PACS-2 under acidic conditions (n=3). (B) Quantitative analysis of SP1 in whole cell, cytoplasm, and nucleus of NPSCs treated with PACS-2 plasmid and control vector under acidic conditions (n=3). (C) Quantitative analysis of p-SP1 (T453), p-SP1 (T739) and total SP1 in NPSCs treated with si-Scr and si-PACS-2 under acidic conditions (n=3). (D) Quantitative analysis of p-SP1 (T453), p-SP1 (T739) and total SP1 in NPSCs treated with PACS-2 plasmid and control vector under acidic conditions (n=3). Data are presented as mean ± SD. ns, not significant, ***p* < 0.01, ****p* < 0.001.


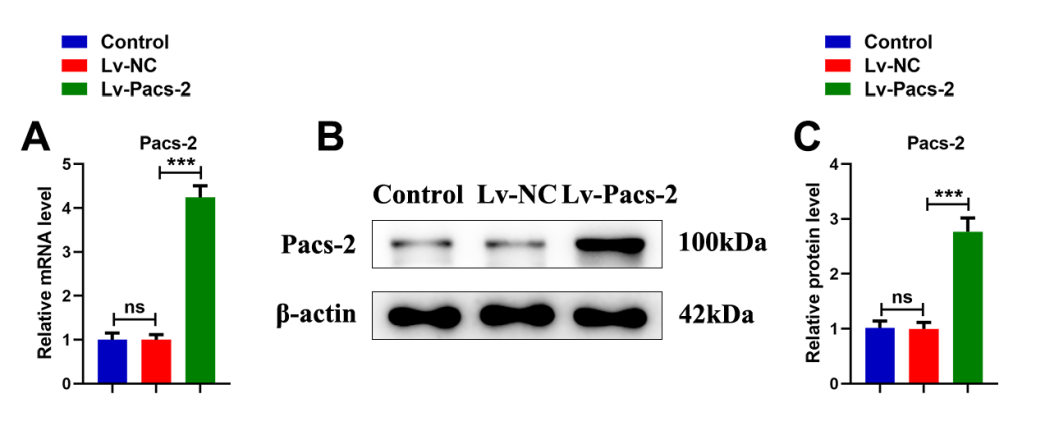


**Figure S6.** (A) qRT-PCR analysis of the mRNA level of Pacs-2 after lentivirus transfection (n=3). (B,C) Western blot analysis of the protein level of Pacs-2 after lentivirus transfection (n=3). Data are presented as mean ± SD. ns, not significant, ****p* < 0.001.

**Supplementary Tables**

**Table S1.** Patient’s information involved in this study.

| No. | Age | Gender | Diagnosis | IVD level | Pfirrmann Grade |
| --- | --- | --- | --- | --- | --- |
| 1 | 62 | female | lumbar disc herniation | L4-L5 | IV |
| 2 | 49 | female | lumbar disc herniation | L4-L5 | IV |
| 3 | 51 | male | lumbar disc herniation | L5-S1 | IV |
| 4 | 59 | male | lumbar disc herniation | L5-S1 | IV |
| 5 | 68 | female | lumbar disc herniation | L5-S1 | IV |
| 6 | 43 | male | lumbar disc herniation | L3-L4 | IV |
| 7 | 61 | female | lumbar disc herniation | L4-L5 | IV |
| 8 | 58 | male | lumbar disc herniation | L4-L5 | IV |
| 9 | 35 | male | lumbar disc herniation | L5-S1 | II |
| 10 | 46 | male | lumbar disc herniation | L4-L5 | II |
| 11 | 39 | female | lumbar disc herniation | L5-S1 | II |
| 12 | 15 | female | vertebral fracture | L2-L3 | II |
| 13 | 48 | male | vertebral fracture | L1-L2 | II |
| 14 | 48 | male | vertebral fracture | L1-L2 | II |
| 15 | 39 | male | vertebral fracture | L1-L2 | II |
| 16 | 38 | male | vertebral fracture | L3-L4 | II |

**Table S2.** The information for antibodies in this study.

| Antibodies | Source | Catalog No. |
| --- | --- | --- |
| PACS-2 | Invitrogen | PA5-100167 |
| Grp78 | Proteintech | 11587-1-AP |
| p-eIF2α | Cell Signaling Technology | 9721 |
| eIF2α | Cell Signaling Technology | 9722 |
| ATF4 | Cell Signaling Technology | 11815 |
| CHOP | Cell Signaling Technology | 2895 |
| Bcl-2 | Proteintech | 26593-1-AP |
| Bax | Proteintech | 50599-2-Ig |
| Cleaved caspase-3 | Cell Signaling Technology | 9664 |
| PGC-1α | Proteintech | 66369-1-Ig |
| SIRT3 | Proteintech | 10099-1-AP |
| FATE-1 | Proteintech | 23809-1-AP |
| LRRK2 | Cell Signaling Technology | 13046 |
| Ubiquitin | Cell Signaling Technology | 3936 |
| Mfn2 | Proteintech | 12186-1-AP |
| JNK | Immunoway | YT2440 |
| p-JNK | Immunoway | YP0157 |
| SP1 | Cell Signaling Technology | 9389 |
| IP3R1 | Proteintech | 19962-1-AP |
| VDAC1 | Proteintech | 66345-1-Ig |
| S*PXR/K | Cell Signaling Technology | 2325 |
| p-SP1 T739 | Immunoway | YP0248 |
| p-SP1 T453 | Immunoway | YP0247 |
| Lamin B1 | Proteintech | 66095-1-Ig |
| β-actin | Proteintech | 20536-1-AP |

**Table S3.** The sequences of siRNA in this study.

| Species | Target | Sequence（5’-3’） |
| --- | --- | --- |
| Human | PACS-2 | GCGACGUCAAGUUCUUCCA |
|  |  | UGGAAGAACUUGACGUCGC |
| Human | LRRK2 | UAGGCUUACAUUAGGUAAUUU |
|  |  | AAAUUACCUAAUGUAAGCCUA |
| Human | Mfn2 | CAGAAGAACAGGUUCUGGACGUCAA |
|  |  | UUGACGUCCAGAACCUGUUCUUCUG |
| Human | SP1 | ACCUGGAGUGAUGCCUAAUAU |
|  |  | AUAUUAGGCAUCACUCCAGGU |

**Table S4.** Primers used in this study

| Species | Gene Name | Sequence（5’-3’） |
| --- | --- | --- |
| Human | LRRK2 | F: TGCGAAGAGGACGAGGAAAC |
|  |  | R: GGAGTACGTGAACACCAGCA |
| Human | Mfn2 | F: CACAAGGTGAGTGAGCGTCT |
|  |  | R: TCCATGTACTCGGGCTCTGA |
| Human | SP1 | F: AGAACCCACAAGCCCAAACA |
|  |  | R: ATGGAAGCAGCTGAGGCAAT |
| Rat | Pacs-2 | F: CCTGGGCTATAAGACGCTGG |
|  |  | R: ATGGTCAATGGGCTGACTGG |
| Human | β-actin | F: ATTGCCGACAGGATGCAGAA |
|  |  | R: CGGACTCGTCATACTCCTGC |
| Rat | β-actin | F: TGCTGACAGGATGCAGAAGG |
|  |  | R: CGGACTCATCGTACTCCTGC |
